# Differentiation therapy with hepatocyte nuclear factor 4α for patients with hepatocellular carcinoma

**DOI:** 10.1038/s41422-025-01142-3

**Published:** 2025-07-04

**Authors:** Chuan Yin, Wen-Ping Xu, Wei-Hua Dong, Chen-Hong Ding, Jia-Rong Cai, Xin Zeng, Si-Han Wu, Pei-Mei Shi, Xin Zhang, Wei-Fen Xie

**Affiliations:** 1 Department of Gastroenterology, Changzheng Hospital, Naval Medical University, Shanghai, China; 2 Department of Interventional Radiology, Changzheng Hospital, Naval Medical University, Shanghai, China; 3 Department of Gastroenterology, Shanghai East Hospital, Tongji University School of Medicine, Shanghai, China

**Keywords:** Drug development, Liver cancer

Dear Editor,

Differentiation therapy is an approach designed to revert tumor cells to a normal cellular state, thereby adopting benign characteristics.^1^ This approach has achieved hallmark success in treating acute promyelocytic leukemia (APL) but has limited effectiveness in solid tumors. This difference in therapeutic efficacy may be due to the genetic complexity of the development of most solid tumors, which involve multiple oncogenic pathways, in contrast to the genetic simplicity of APL development, which is predominantly driven by PML-RARα.^2^ Transcription factors (TFs) recognize specific DNA motifs in the genome to regulate gene expression and determine cell fates, with the upregulation of a single TF capable of triggering significant cellular transformations.^3^ Hepatocyte nuclear factor 4α (HNF4α) is a master TF regulating hepatocyte differentiation.^4^ Restoring HNF4α expression induces the differentiation of hepatocellular carcinoma (HCC) cells into mature hepatocytes and has demonstrated significant therapeutic efficacy in various HCC animal models.^5,6^ Lipid nanoparticles (LNPs), widely employed for RNA delivery, offer hepatocyte specificity, effectively minimizing systemic exposure and toxicity. Self-replicating RNA (srRNA), with the self-amplifying nature, allows for a lower RNA dose for the treatment.^7^ A recent study reported that systemic injection of LNP-encapsulated srRNA achieves significantly prolonged expression of the target gene specifically within tumors but not in normal organs of mice.^8^ These characteristics make LNP-encapsulated srRNA an ideal candidate for clinical therapy in HCC. However, to date, no studies have been reported on the use of srRNA for the treatment of human cancers. In this study, we reported the development and preclinical evaluation of CD-801, a LNP-encapsulated srRNA encoding HNF4α. A subsequent first-in-human, first-in-class, dose-escalation trial was conducted to assess the safety, tolerability, and efficacy of CD-801 in patients with advanced HCC.

Firstly, *HNF4α* srRNA and a control srRNA were developed by re-engineering the Venezuelan Equine Encephalitis Virus (VEEV) genome from the Alphavirus family, where the structural protein-coding gene was replaced with *HNF4α* or *GFP*. *HNF4α* srRNA and the control srRNA were subsequently assembled with LNP to generate *HNF4α* srRNA-LNP (CD-801) and *GFP* srRNA-LNP (*GFP* srRNA), respectively (Supplementary information, Data S1). Real-time PCR revealed that Huh7 HCC cells demonstrated more robust and sustained HNF4α expression compared to that observed in primary human hepatocytes in vitro. CD-801 delivery upregulated the expression of HNF4α protein up to 7 days in HCC cells and inhibited the HCC malignancy, accompanied by enhanced hepatic function and decreased expression of stemness markers in vitro (Supplementary information, Fig. S1). We then used two HCC xenograft models to evaluate the therapeutic effect of CD-801 in vivo. Intratumoral injection of CD-801 resulted in enhanced expression of HNF4α protein and characteristic hepatocyte marker genes (including *ALDOB*, *APOCIII*, *PCK2*, *GYS2*, and *BR*), and significant suppression of tumor growth, weight, and Ki67 expression in the subcutaneous HCC xenograft models (Supplementary information, Fig. S2). To further investigate whether systemic administration of CD-801 exerts a similar anti-tumor effect, we detected the distribution of HNF4α expression across various organs and tumor tissues following a single dose of CD-801 administration via the tail vein in the orthotopic HCC xenograft models. Real-time PCR and ELISA showed that srRNA-mediated HNF4α expression accumulated in the heart, liver, spleen, lung, kidney, and tumor as soon as 2 h after CD-801 delivery. Then HNF4α level declined rapidly in normal tissues but increased gradually in tumors (Supplementary information, Fig. S3a, b). Western blotting and immunohistochemistry analysis showed that HNF4α protein expression peaked on day 3 and remained elevated until day 7 in CD-801-treated tumor tissues (Supplementary information, Fig. S3c, d). The upregulation of HNF4α increased hepatocyte-specific gene expression and concurrently induced senescence and apoptosis in these tissues (Supplementary information, Fig. S3e, f). Moreover, administration of two doses of CD-801 via the tail vein, at a 7-day interval, also resulted in a significant reduction in size, weight, and Ki67 expression in tumor nodules, compared to the saline or *GFP* srRNA groups in the orthotopic HCC xenograft models (Supplementary information, Fig. S3g, l).

Acute toxicity assessment showed that CD-801 administration via intravenous injection of a single dose of 150 µg/animal had no CD-801-related toxic effects in rats (Supplementary information, Data S2).

Then, this single-center, open-label, dose-escalation study evaluated the safety, tolerability, and efficacy of CD-801 in patients with advanced HCC enrolled in Shanghai Changzheng Hospital between June 30, 2023, and August 28, 2023. All cases were unresectable, and the patients either were not eligible for or no longer benefited from locoregional or systemic therapy (Supplementary information, Fig. S4). The protocol was approved by the institutional ethics committee (No. 2023SL036) of Shanghai Changzheng Hospital and registered on https://www.chictr.org.cn (ChiCTR2300073093). A total of 9 male patients with advanced HCC, aged between 43 and 74 years, were sequentially enrolled into three dose groups, with each group consisting of 3 participants (Supplementary information, Table S1). All patients had a diagnosis of HBV infection and were undergoing long-term oral administration of nucleos(t)ide analogs. The patients were categorized as Barcelona Clinic Liver Cancer stage B (*n* = 3) or C (*n* = 6) and China liver cancer stag stage II (*n* = 3) or III (*n* = 6). With the exception of patient 4, who is ineligible for locoregional or systemic therapy, the remaining eight patients had undergone more than 2 types of anti-cancer therapies prior to enrollment. At baseline, 8 of the 9 subjects exhibited elevated AFP levels, with 7 exceeding 400 ng/mL (including 2 cases above the detection limit of 60,500 ng/mL).

As of the cutoff date for the analysis on August 20, 2024, the patients received 2‒13 doses of CD-801 administration and were followed up for a duration ranging from 6.6 to 58.6 weeks, with a median follow-up of 26 weeks (Supplementary information, Fig. S5). Three patients (patients 1, 6, and 7) discontinued the trial due to disease progression after 2‒5 doses of CD-801 delivery. The remaining patients received at least 5 doses of CD-801, culminating in the achievement of the prescribed objective dosage of 100 μg. All patients completed the 2-week dose-limiting toxicities (DLT) observation period, during which no DLT events were observed. The maximum tolerated dose was not reached.

All participants encountered one or more adverse events, regardless of the dosage administered (Supplementary information, Table S2). Most of these adverse events were mild and temporary, either self-limiting without any intervention or showing quick improvement with symptomatic treatment. Specifically, 3 patients experienced grade 1 fever on the second day of treatment, and 1 patient developed grade 1 fatigue on the treatment day. Eighteen grade 3 or 4 adverse events were observed (Supplementary information, Table S2). Of these events, 14 were potentially associated with CD-801 treatment, with most occurring in patients 4, 5, and 9. Five serious adverse events (SAEs), namely tumor hemorrhage, ascites, cytokine release syndrome (CRS), ileus, and elevated total bilirubin levels, were potentially related to treatment (Supplementary information, Table S3). Particularly, patient 5 experienced CRS manifested as a sustained fever peaking at 39.5 °C, commencing at 6 weeks after initial treatment, followed by impaired liver function, anorexia, and malaise, coupled with elevated biomarker levels such as interleukin-6 and C-reactive protein (Supplementary information, Fig. S6). The SAEs in patients 4 and 9 occurred at least 7 weeks following the final administration of CD-801. Moreover, six patients exhibited mild HBV reactivation, evident by a rise in HBV DNA levels varying from 238 to 13, 900 copies/mL. This reactivation occurred between 13.4 and 26.4 weeks after initiating CD-801 treatment and was controlled through the combination therapy of Entecavir and Tenofovir. As of the cutoff date, 6 patients had deceased across three study doses. These deaths occurred within a range of 39‒155 days following the last treatment. The corresponding treatment doses and the specific causes of death were detailed in Supplementary information, Table S4.

The cumulative survival rate for all nine patients was 88.9% at 3 months, 55.6% at 6 months and 33.3% at 12 months (Supplementary information, Fig. S7). As of the latest data collection, 3 patients were still under follow-up. Based on the mRECIST criteria assessed by MRI scans, no patients achieved a complete or partial response. However, patients 4 and 8 maintained stable disease, with follow-up durations of 40 and 53 weeks, respectively (Fig. 1; Supplementary information, Fig. S8). It is noted that the liver lesion in patient 8 progressed, accompanied by a marked elevation of AFP levels before enrollment, but the lesion remained stable up to 53 weeks following CD-801 treatment (Supplementary information, Fig. S8). At 24 weeks following the initial treatment with CD-801, patient 4 demonstrated a decrease in arterial phase enhancement within the liver tumor, suggesting a reduction in the viable tumor components. This area of reduced enhancement continued to gradually expand up to 37 weeks. Furthermore, distinct focal necrosis in his liver tumor was observed beginning at 30 weeks (Fig. 1a‒f). Concurrently, the patient’s pulmonary metastatic lesion shrank starting at 18 weeks following treatment initiation, further diminishing at 24 weeks, and becoming undetectable by 30 weeks (Fig. 1g‒k). Consistent with these radiological findings, the pathological examination of the liver lesion from patient 4 revealed that the majority of the sample was dominated by severe fibrosis at 24 weeks after initial infusion, and severe necrosis was also observed at 33 weeks. Significantly, the samples revealed substantial inflammation, with a notable presence of CD8^+^ and CD4^+^ T cells in the tumor tissues (Fig. 1l‒p).

**Fig. 1:**
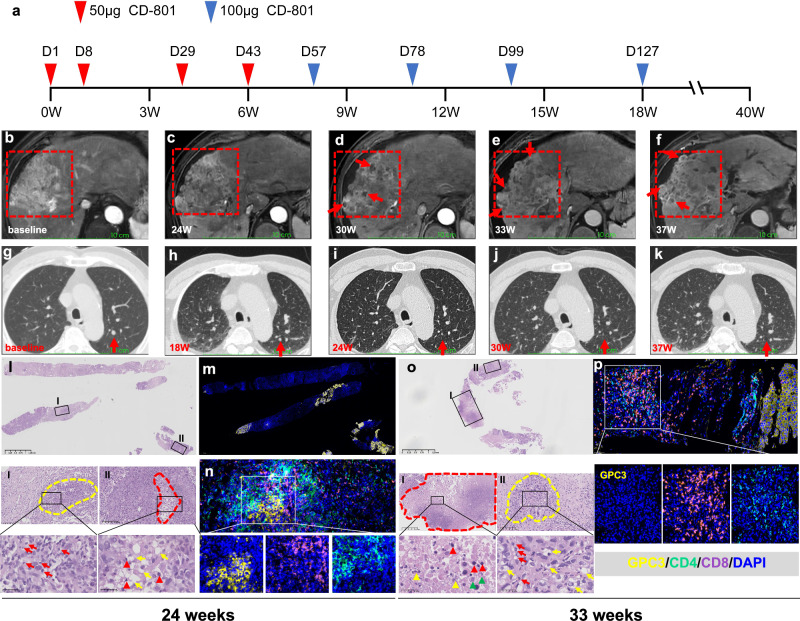
Responses of patient 4 to CD-801 therapy. **a** A schematic representation of CD-801 treatment for patient 4. **b** The contrast-enhanced MRI image of target lesion (red dashed line) at baseline. **c**‒**f** The target lesion size remained largely stable but showed a gradual reduction of arterial phase enhancement after the initial CD-801 treatment. The necrosis was clearly observed starting at 30 weeks (red arrows) (**d**‒**f**). **g**‒**k** The chest CT images of the pulmonary metastatic nodule (red arrow), which began to regress at week 18 (**g**, **h**) and was undetectable at 30 and 37 weeks after treatment (**j**, **k**). **l**‒**n** The examination of liver cancer biopsies from patient 4 at 24 weeks post-initial treatment. H&E staining showed that extensive fibrotic tissue was present throughout the entire liver biopsy specimen (**l**), with severe inflammatory cell infiltration (red arrows) within the residual tumor nests (yellow dashed line, I) and nuclear pyknosis (red arrowhead) and hydropic change (yellow arrows) of degenerated tumor cells in the focal necrosis (red dashed line, II). Immunofluorescence staining exposed only a few isolated small cancer nests within the entire tissue section (**m**). Multiplex immunofluorescence revealed substantial infiltration of CD4^+^ (green) and CD8^+^ (purple) T cells around the remaining GPC-3-positive tumor cells (yellow) (**n**). **o**, **p** The examination of liver cancer biopsies from patient 4 at 33 weeks post-initial treatment. H&E staining showed extensive fibrotic tissues and coagulative necrosis (red dashed line) throughout the entire liver biopsy specimen (**o**). Nuclear pyknosis (red arrowhead), karyorrhexis (yellow arrowheads), and lysis (green arrowheads) were detected in the necrotic tissues (**I**). Pronounced infiltration of inflammatory cells (red arrows) and hydropic degeneration of tumor cells (yellow arrows) were observed within the tumor nest (yellow dashed line, II). Multiplex immunofluorescence showed robust infiltration of CD4^+^ and CD8^+^ T cells within fibrotic tissue (**p**).

It is noteworthy that 4 participants (patients 2, 3, 5, and 9) who were evaluated as having progressive disease exhibited distinct profiles in MRI and histology. The target lesion in patient 3 remained largely stable until 18 weeks after the initiation of therapy but showed slight enlargement at 48 weeks. However, there was a gradual increase in the reduced enhancement area in the arterial phase starting at 18 weeks post-treatment, which on biopsy showed an escalating collagen deposition and progressive decrease of tumor clusters. The remaining tumor tissue showed evidence of hydropic degeneration in tumor cells, accompanied by a robust infiltration of inflammatory cells, predominantly characterized by the presence of CD8^+^ and CD4^+^ T cells at 48 weeks (Supplementary information, Fig. S9). Similar tumor enlargement coupled with decreased perfusion following CD-801 treatment was also observed in patients 2, 5, and 9. Specifically, in patient 2, both the lesions in the right posterior lobe and the left medial lobe of the liver had increased in size compared to baseline at 32 weeks post-treatment, but it remained stable from then until week 53, with a decline in arterial phase enhancement at the center during treatment (Supplementary information, Figs. S10a‒f, S11a‒e). Microscopic examination of the lesion in the left medial lobe revealed progressive collagen deposition within the tumor, contrasting with the absence of collagen in the adjacent liver tissues from 32 weeks to 49 weeks post-treatment. In parallel, the residual tumor tissue experienced a significant reduction, accompanied by clear signs of hydropic degeneration in the tumor cells and a moderate influx of inflammatory cells, particularly at 49 weeks (Supplementary information, Fig. S10g‒o). Consistently, pathological examination with the lesion in the right posterior lobe of the liver also revealed excessive collagen deposition which infiltrated the residual cancer nests at 32 weeks post-treatment (Supplementary information, Fig. S11f). In patient 5, MRI images indicated slight enlargement of tumor lesions 1 week after CD-801 administration, showing numerous necrotic cavities of varying sizes within the tumors at 6 weeks post-treatment, partly surrounded by a thin, uniform peripheral enhancement (Supplementary information, Fig. S12a‒i). In patient 9, the target tumor also initially expanded slightly at 2 weeks and continued to enlarge with a progressive decrease in arterial enhancement within the entire lesion by 17 weeks after therapy. This pattern of tumor enlargement and diminishing enhancement persisted for at least 8 weeks after treatment cessation (Supplementary information, Fig. S12j‒n).

The serum AFP levels exhibited notable variations among the patients. In patient 8, AFP levels declined to within the normal range (0–20 ng/mL) after 6 weeks of treatment and remained marginally above the range thereafter. Five patients displayed a rapid or fluctuating elevation in serum AFP levels throughout the entire observation period (Supplementary information, Fig. S13).

In this first-in-class, first-in-human study, the resulting data suggested that CD-801 was safe, well-tolerated, and demonstrated promising therapeutic efficacy in advanced HCC patients. It has been reported that HNF4α augments T-cell function and bolster anti-tumor immunity by reducing ammonia levels.^9^ In this study, we also found a significant infiltration of CD4^+^ and CD8^+^ T cells within the tumors upon the treatment, suggesting that differentiation therapy with HNF4α may reprogram the tumor immune microenvironment. It should be highlighted that in three cases, SAEs potentially related to treatment emerged more than 7 weeks after the last dose of CD-801. This delay in onset might be ascribed to the enduring activation of a tumor-specific immune response and cytokine release in certain HCC patients. While the majority of these adverse events can be effectively alleviated through corticosteroid therapy in line with the guidelines for immune-related adverse events, close monitoring of hepatic function, cytokine profiles, and clinical symptoms remains essential throughout the treatment course.

In our study, HBV reactivation occurred in 6 of 9 patients. Dual antiviral therapy with entecavir and tenofovir stabilized their viral loads at low levels (range: 238‒13,900 copies/mL). Despite no observed impact on liver function, the long-term outcomes of HBV reactivation remain uncertain due to limited follow-up. We advocate for standardized prophylactic antiviral therapy for all HBsAg-positive patients, regardless of baseline HBV DNA levels, in line with current HCC immunotherapy guidelines. Notably, in addition to severe infiltration of CD4^+^ and CD8^+^ T cells, distinct intra-tumoral necrosis and fibrotic tissue repair were also observed in all three subjects (patients 2, 3, 4) who underwent liver biopsy. Their tumors exhibited enlargement coupled with hypovascularity as seen on MRI scans after CD-801 treatment. This type of tumor response closely aligns with the characteristics of pseudoprogression (PsPD), a phenomenon induced by immunotherapy,^10^ suggesting PsPD in these patients. Further investigation is necessary to uncover the true outcome profiles of this therapy. In conclusion, this proof-of-concept investigation validates our previous hypothesis that differentiation therapy utilizing differentiation-determining TFs may represent an effective strategy for the treatment of human malignancies.^6^

## Supplementary information

merged supplementary file
