# Supplementary material for: Differentiation therapy with hepatocyte nuclear factor 4α for patients with hepatocellular carcinoma
Source: Cell Res. 2025 Jul 4;35(9):687–90. doi: 10.1038/s41422-025-01142-3 (PMC12408822; doi:10.1038/s41422-025-01142-3)
Supplement: Supplementary file 1 — merged supplementary file [file 41422_2025_1142_MOESM1_ESM.pdf]

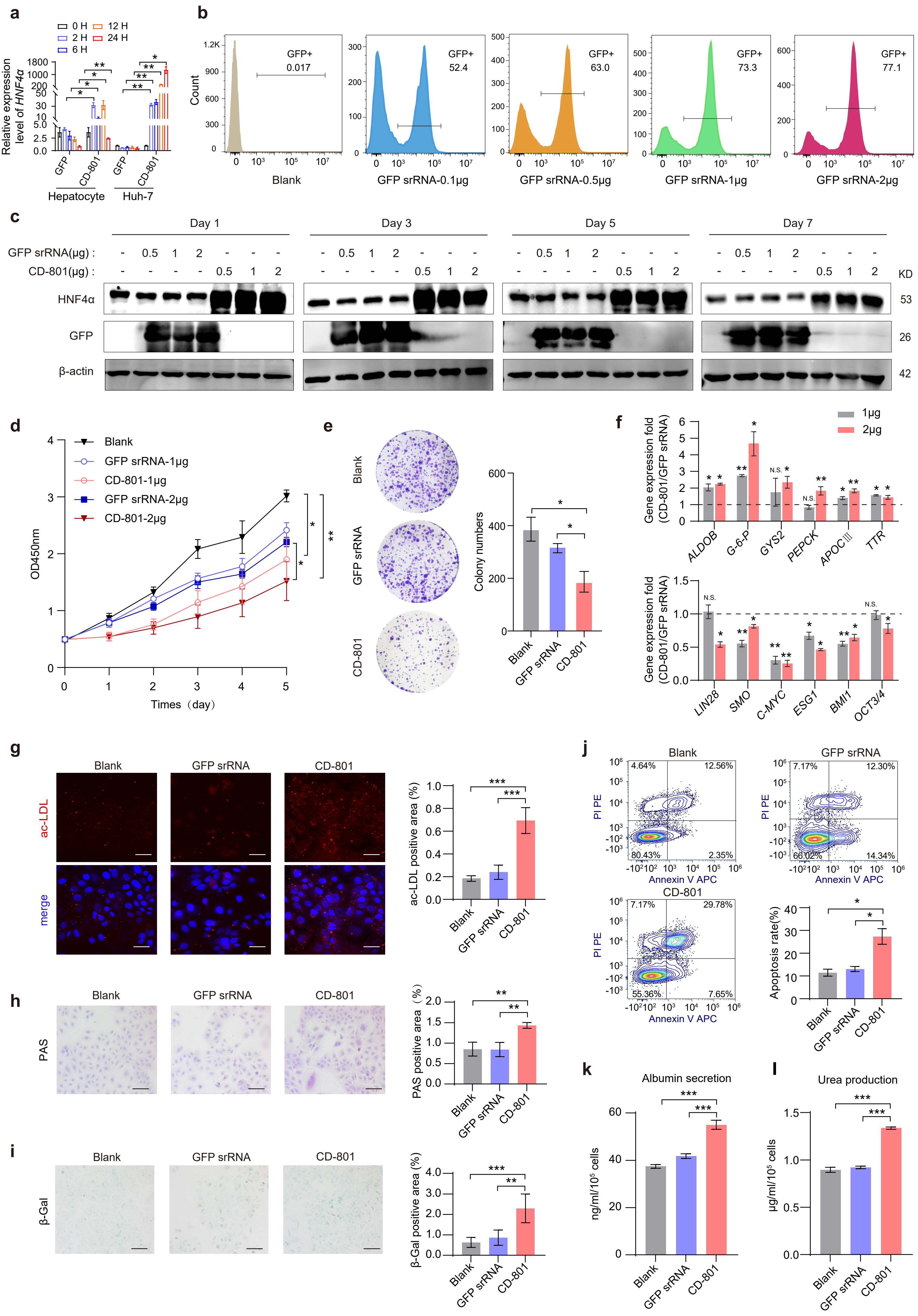


**Figure S1. Anti-HCC effect of CD-801 *in vitro.***

**a** The RNA levels of human HNF4α in Huh-7 cells and normal primary human hepatocytes were measured at 2, 6, 12, and 24 hours post-treatment with CD-801 using RT-PCR. **b** Detection for the GFP fluorescence in Huh-7 treated with different doses of GFP srRNA for 24 hours. Experiments were performed in triplicate. **c** Western blotting analysis for expression of HNF4α in Huh-7 cells treated with CD-801 for 1, 3, 5, and 7 days. Experiments were performed in triplicate. **d** The proliferation of Huh-7 cells was measured with the Cell Counting Kit-8. Experiments were performed in triplicate. **e** Colony formation assays. Experiments were performed in triplicate. **f** RT-PCR analysis for the expression of characteristic hepatocyte markers and stemness markers in CD-801 treated Huh-7 cells. Experiments were performed in triplicate. *ALDOB*, aldolase B; *G-6-P,* glucose-6-phosphatase; *GYS2*, glycogen synthetase 2; *PEPCK*, phosphoenolpyruvate carboxykinase; *APOCIII*, apolipoprotein C III; *TTR*, transthyretin; *LIN28*, lin-28 homolog A; *SMO*, smoothened, frizzled class receptor; *C-MYC*, MYC proto-oncogene, bHLH transcription factor; *ESG1*, embryonic cell-specific gene 1; *BMI1*, BMI1 proto-oncogene, polycomb ring finger; *Oct3/4*, POU class 5 homeobox 1. **g** ac-LDL uptake ability of Huh-7 cells was analyzed using the Dil-ac-LDL fluorescent substrate (red) (left). The ac-LDL positive area was quantified by Image-Pro Plus 6.0 software (right). Scale bar = 25 μm. Experiments were performed in triplicate. **h** PAS staining was used to detect the effect of CD-801 on the stored glycogen in Huh-7 cells (left). The PAS positive area was quantified by Image-Pro Plus 6.0 software (right). Scale bar = 50 μm. Experiments were performed in triplicate. **i** Representative images of β-galactosidase activity staining of Huh-7 cells (left). The β-gal positive cells were quantified by Image-Pro Plus 6.0 software (right). Scale bar = 50 μm. Experiments were performed in triplicate. **j** Apoptosis in Huh-7 cells was assessed using FACS. Experiments were performed in triplicate. **k** Alb secretion in Huh-7 cells was measured by ELISA. Experiments were performed in triplicate. **l** Urea production derived from Huh-7 cells was analyzed using the QuantiChrom™ Urea Assay Kit. Experiments were performed in triplicate. Data were presented as means±SEM. A one-way ANOVA (d, e, g, h, i, j, k, l) or Student's t-test (a, f) was used to analyze the differences between two groups. * $P < 0.05$, ** $P < 0.01$, *** $P < 0.001$, and "N.S." indicates no significant difference.

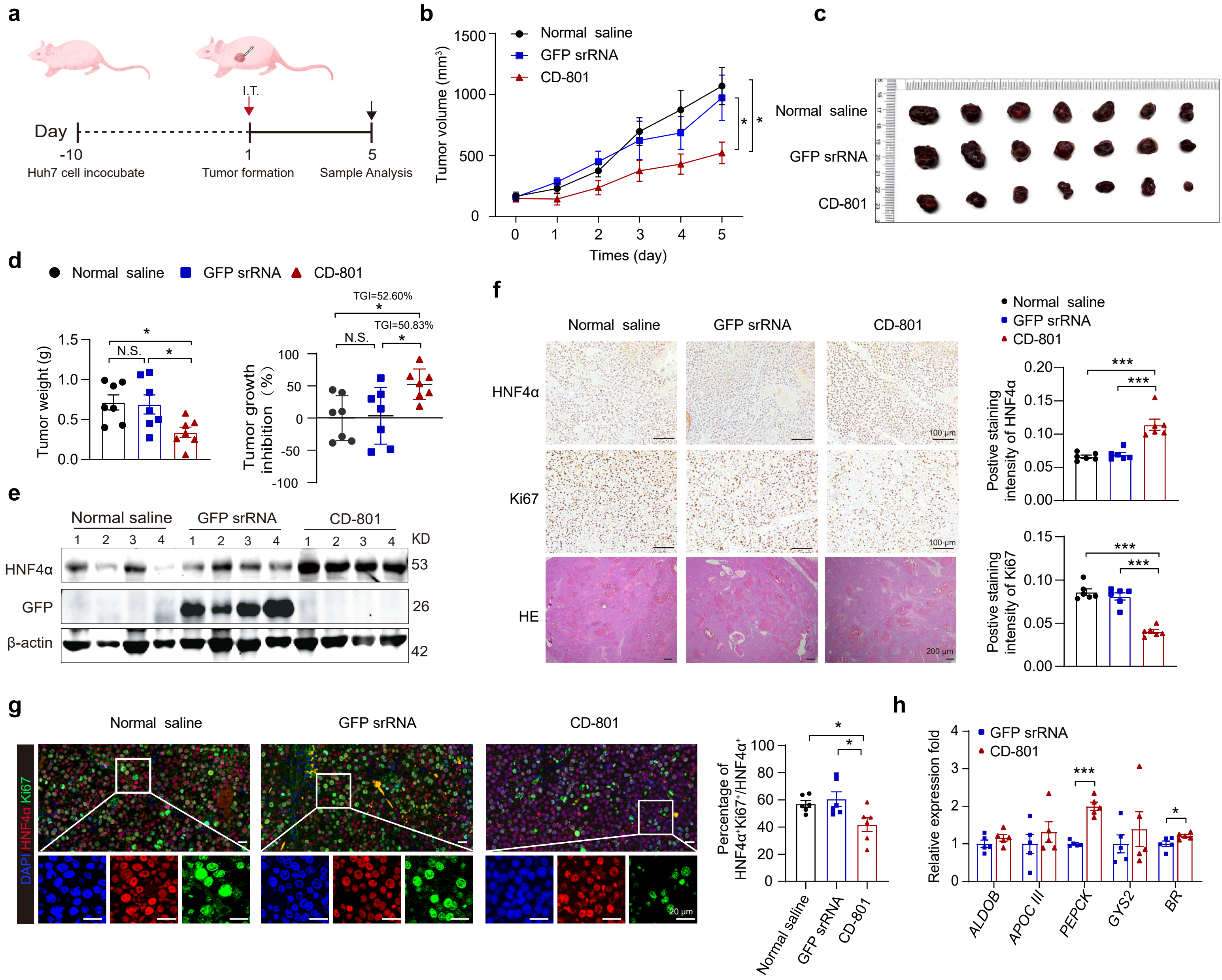


**Figure S2. Anti-HCC effect of CD-801 on subcutaneous HCC xenograft model.**

**a** Schematic representation of the CD-801 therapy experiment. The Huh-7 xenografts were intratumorally injected with normal saline, GFP srRNA, or CD-801, respectively. The subcutaneous HCC xenograft model with CD-801 treatment was performed in triplicate. **b** Tumor growth curve of the subcutaneous Huh-7 xenografts (n = 7 mice/group). **c** Tumors of the subcutaneous Huh-7 xenografts. **d** The tumor weight and growth inhibition of CD-801 treated Huh-7 xenografts compared with that of GFP srRNA treated xenografts. **e** Western blotting for the expression of HNF4α in tumors. **f** Representative images of HE staining and immunohistochemistry staining of HNF4α and Ki67 on tumor sections (left). The positive staining intensity of HNF4α and Ki67 were quantified by Image-Pro Plus 6.0 software (right). The scale bar for HE staining is 200 μm, and for immunohistochemistry staining, it is 100 μm. **g** Representative images of immunofluorescence staining of HNF4α and Ki67 on Huh-7 subcutaneous tumor sections (left). The proportion of $Ki67^{+}HNF4\alpha^{+}$ double-positive tumor cells among total $HNF4\alpha^{+}$ tumor cells was quantified using Image-Pro Plus 6.0 software (right). Scale bar = 20 μm. **h** RT-PCR analysis for the expression of characteristic hepatocyte markers in Huh-7 subcutaneous tumors. Data were presented as means±SEM. A one-way ANOVA (b, d, f, g) or Student’s t-test (h) was used to analyze the differences between two groups. * $P < 0.05$, ** $P < 0.01$, *** $P < 0.001$, and “N.S.” indicates no significant difference. Each point in the bar plot represented the data collected from an individual mouse.

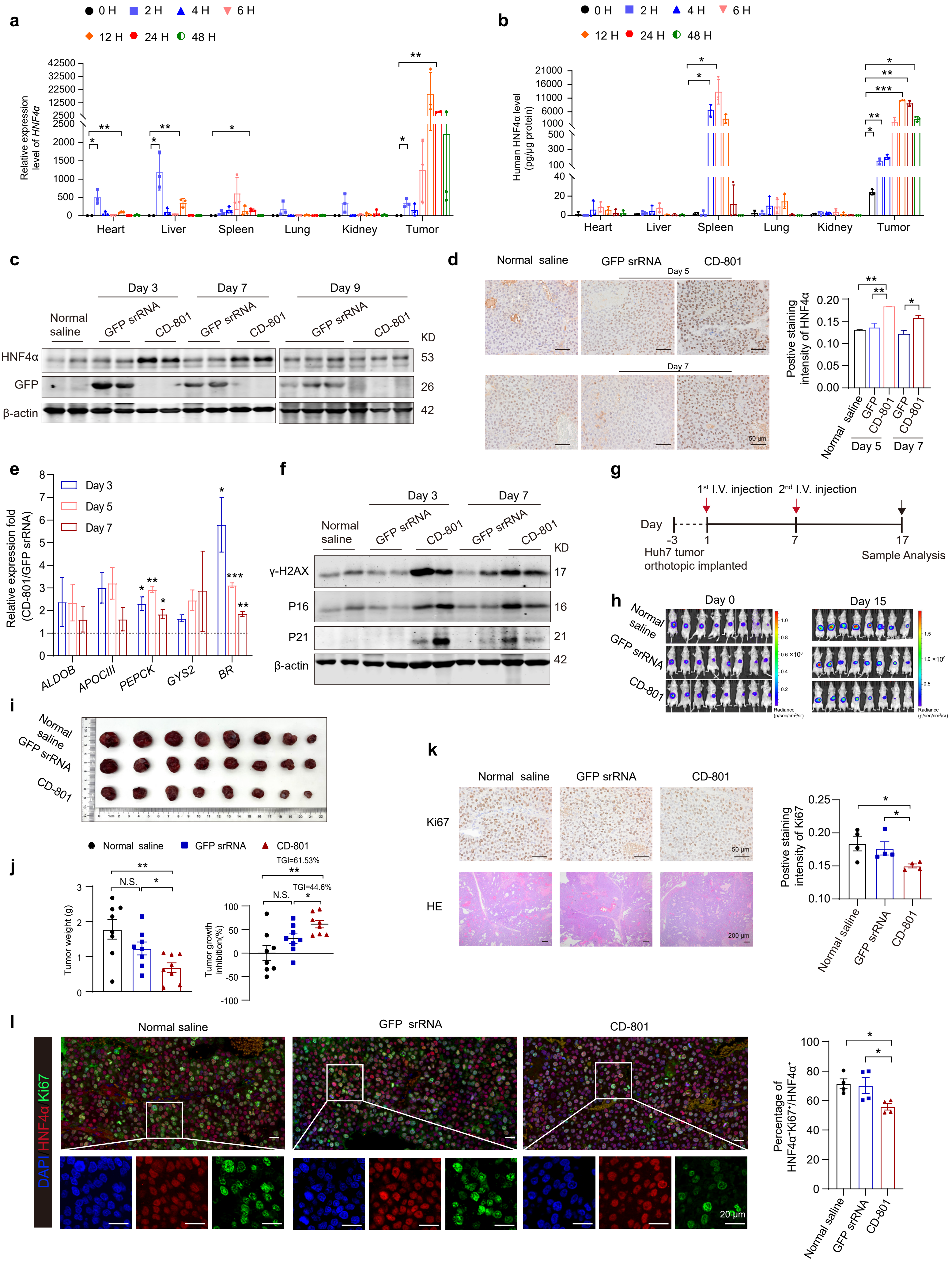


**Figure S3. Anti-HCC effect of CD-801 on orthotopic HCC implantation model.**

**a-b** Mice bearing orthotopic Huh-7 tumors were intravenously injected with CD-801, and organs and tumor tissues were collected at various time points post-treatment. The RNA levels of srRNA-expressed human HNF4α in different organs and tumor tissues were measured at 2, 4, 6, 12, 24 and 48 hours post-treatment with CD-801 using RT-PCR (a). The HNF4α (srRNA) primer was employed for the detection of srRNA-expressed human HNF4α. The protein levels of human HNF4α in different organs and tumor tissues were analyzed by ELISA (b). n = 2 or 3 mice per time point. **c** The HNF4α expression in the orthotopic tumor tissues collected at 3, 7 or 9 days post CD-801 treatment was detected by Western blotting (n = 2 or 3 mice per time point). **d** The IHC staining of HNF4α in the orthotopic tumor tissues collected at 5 or 7 days post CD-801 treatment (left). The positive staining intensity of HNF4α was quantified by Image-Pro Plus 6.0 software (right). n = 2 mice/time point. Scale bar = 50 μm. **e** The expression of characteristic hepatocyte marker genes in the orthotopic tumor tissues collected at 3, 5 or 7 days post CD-801 treatment was analyzed by RT-PCR (n = 2 mice/time point). *ALDOB*, aldolase B; *APOCIII*, apolipoprotein C III; *PEPCK*, phosphoenolpyruvate carboxykinase; *GYS2*, glycogen synthetase 2; *BR*, biliverdin reductase. **f** The levels of senescence marker (P16, P21), and apoptosis marker (γ-H2AX) in the orthotopic tumor tissues collected at 3 and 7 days post CD-801 treatment was detected by Western blotting (n = 2 mice/time point). **g** Timeline of tumor implantation and treatment schedule in the orthotopic HCC model. The mice bearing orthotopic Huh-7 expressing luciferase tumor were administered normal saline, GFP or CD-801 every 7 days via intravenous injection for a total of two injections.The orthotopic HCC model with CD-801 treatment was conducted twice. **h** Bioluminescence images of the luciferase-expressing Huh-7 tumors in mice before and after treatment were shown (n = 8 mice/group). **i** The image of tumors derived from xenograft mouse model. **j** Tumor weight and tumor growth inhibition were analyzed in the xenograft mouse model (n = 8 mice/group). **k** Representative images of HE staining and IHC staining of Ki67 in the Huh-7 orthotopic tumor sections (left). The positive staining intensity of Ki67 were quantified by Image-Pro Plus 6.0 software (right). The scale bar for HE staining is 200 μm, and for immunohistochemistry staining, it is 50 μm. **l** Representative images of immunofluorescence staining of HNF4α and Ki67 in Huh-7 orthotopic tumor sections (left). The proportion of Ki67$^+$HNF4α$^+$ double-positive tumor cells among total HNF4α$^+$ tumor cells were quantified by Image-Pro Plus 6.0 software (right). Scale bar = 20 μm. Data in this figure were presented as means±SEM. A one-way ANOVA (a, b, d, j, k, l) or Student's t-test (e) was used to analyze the differences between two groups. * $P < 0.05$, ** $P < 0.01$, *** $P < 0.001$, and "N.S." indicates no significant difference. Each point in the bar plot represented the data collected from an individual mouse.

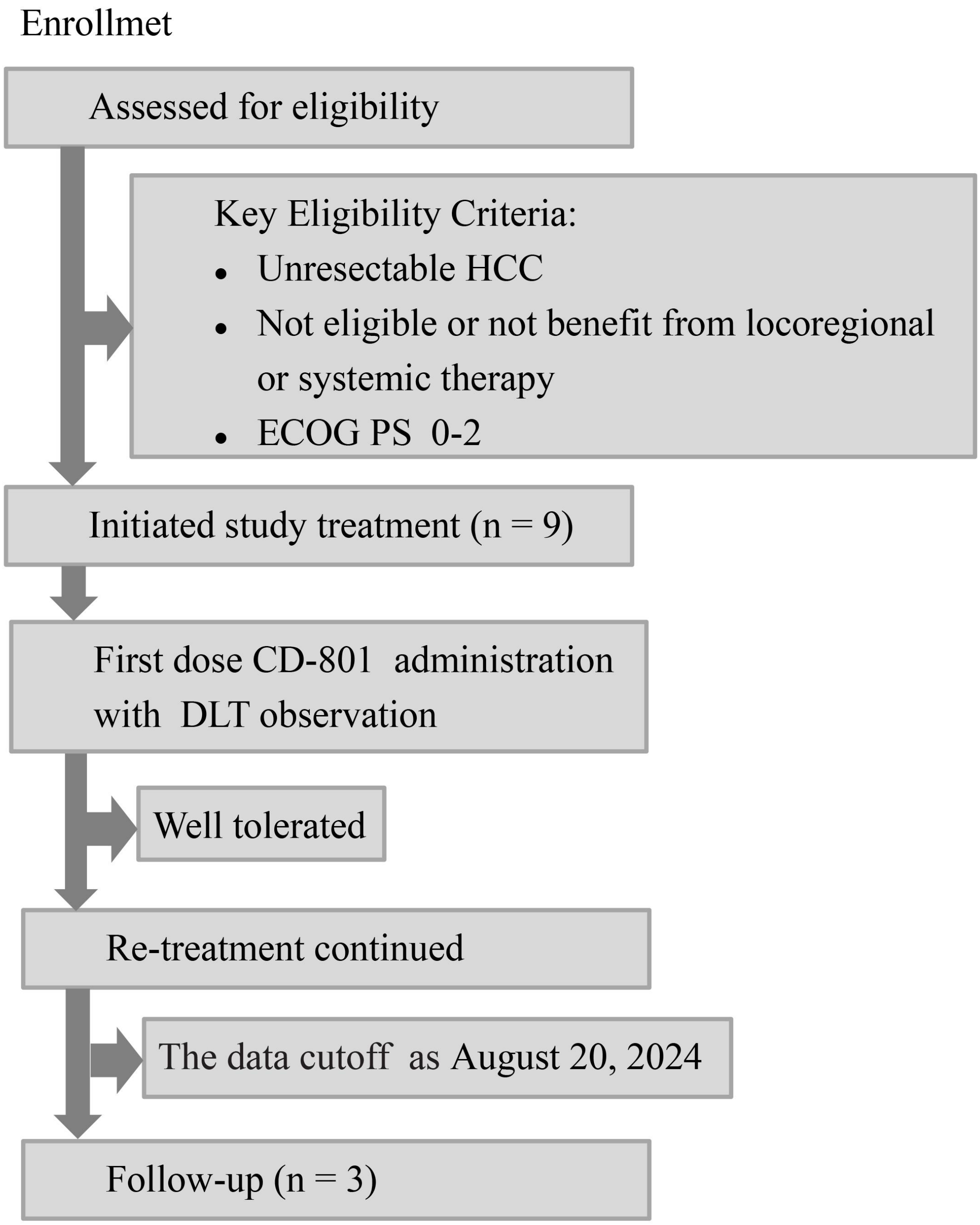


**Figure S4. Patient flowchart.** The diagram shows the flow of patients as of August 20, 2024. HCC, hepatocellular carcinoma; ECOG PS, Eastern Cooperative Oncology Group performance status; DLT, dose-limiting toxicities.

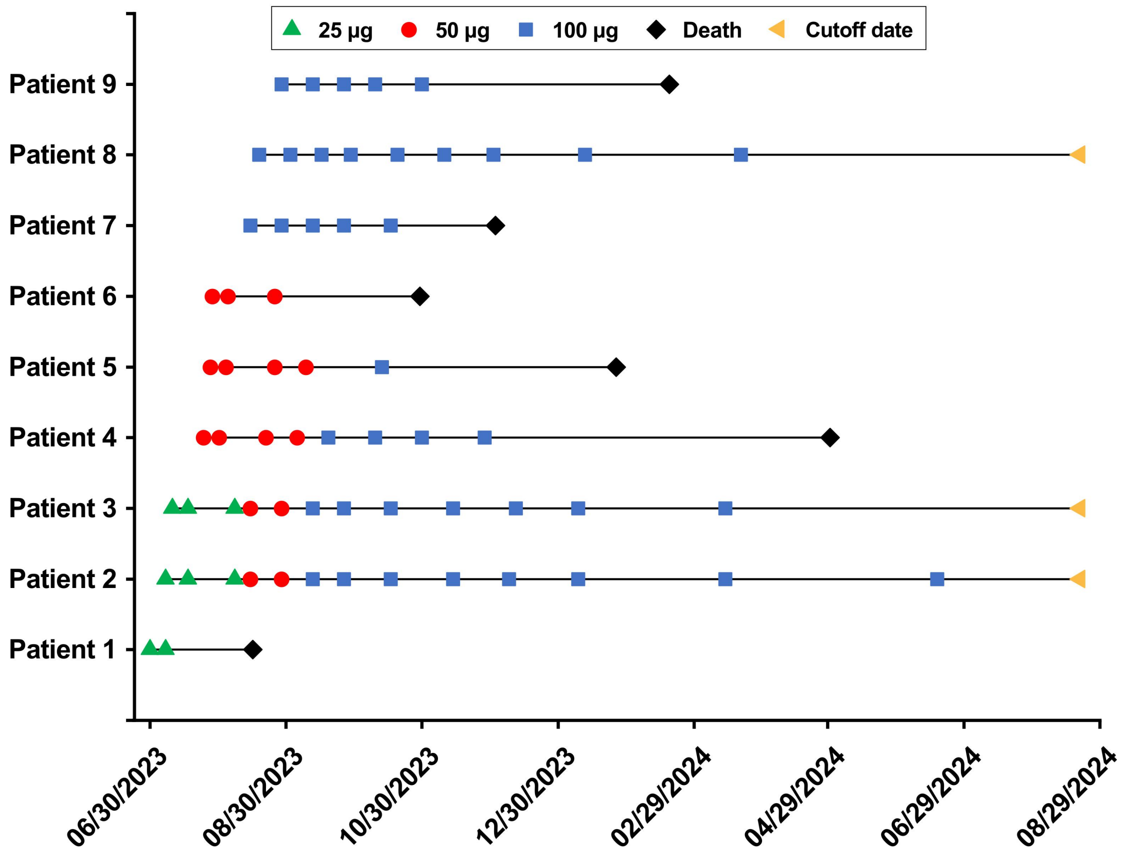


**Figure S5. A schematic representation of CD-801 treatment for each enrolled patient.**

The cutoff date for the analysis was August 20, 2024.

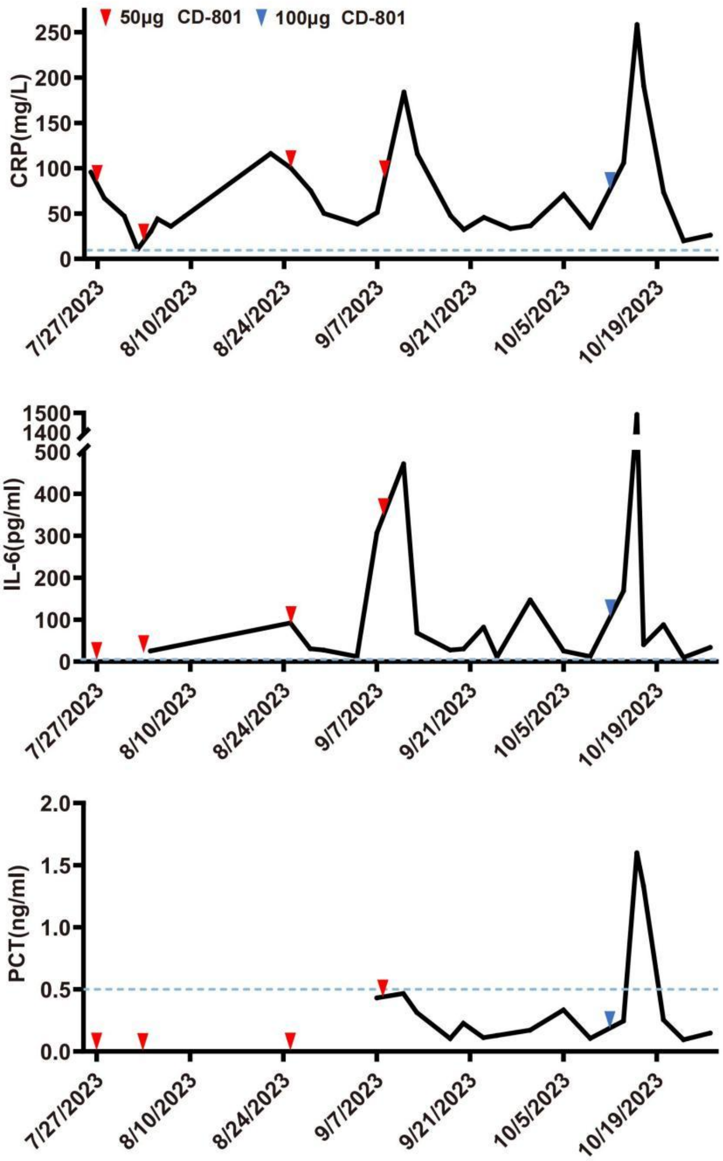


**Figure S6. Changes of serum CRP, PCT, and IL-6 levels in patient 5 throughout CD-801 treatment.** CRP, C-reactive protein; PCT, procalcitonin; IL-6, interleukin-6.

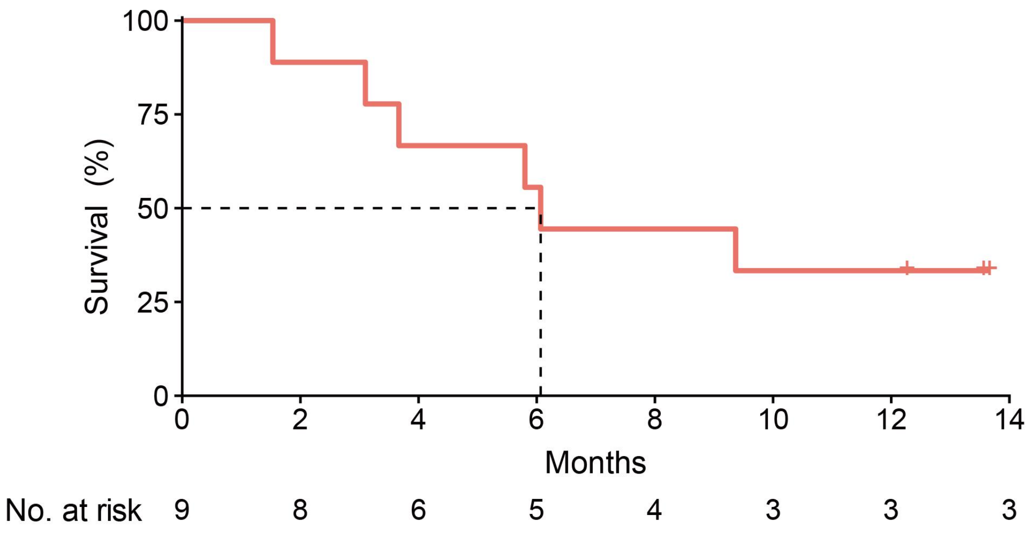


**Figure S7. Kaplan-Meier Analysis for the cumulative survival of all patients.**

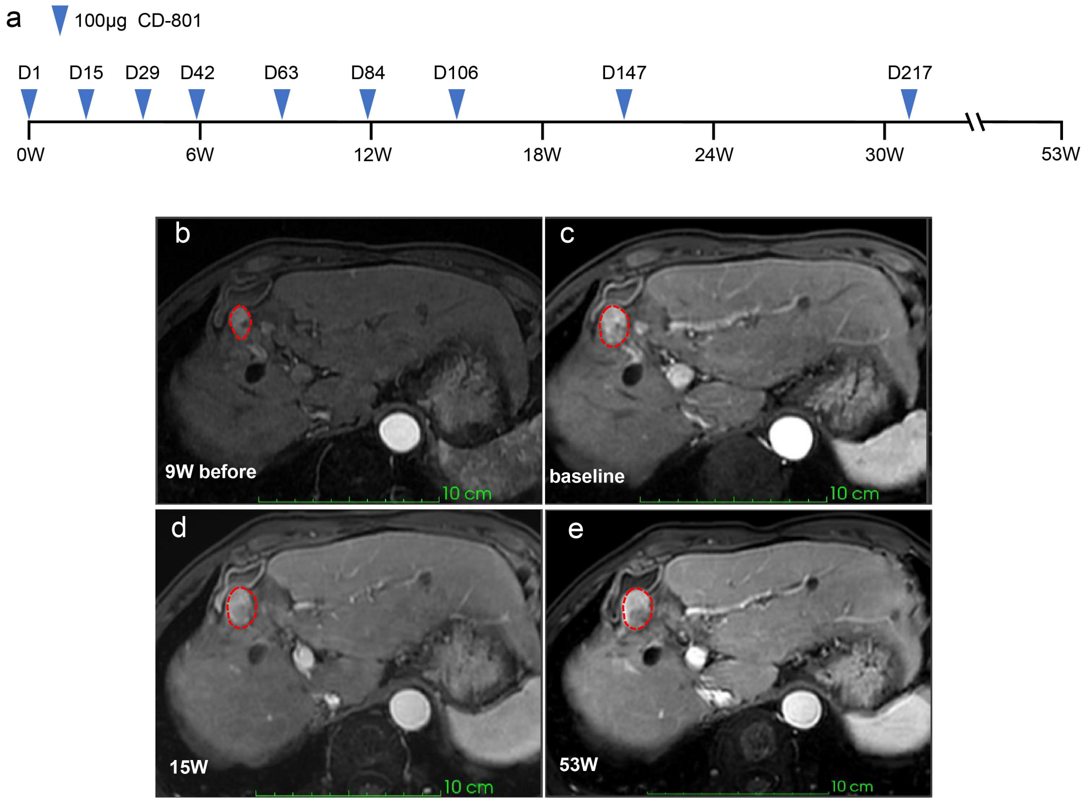


**Figure S8. Responses of patients 8 to CD-801 therapy.**

**a** A schematic representation of CD-801 treatment for patient 8. **b-e** Representative contrast-enhanced MRI images of the target lesion (outlined in red dashed line) in patient 8. The liver lesion, observed 9 weeks prior to study enrollment **(b)**, had notably expanded by the time of the baseline evaluation **(c)**. The lesion remained stable up to 53 weeks after the treatment **(d, e)**.

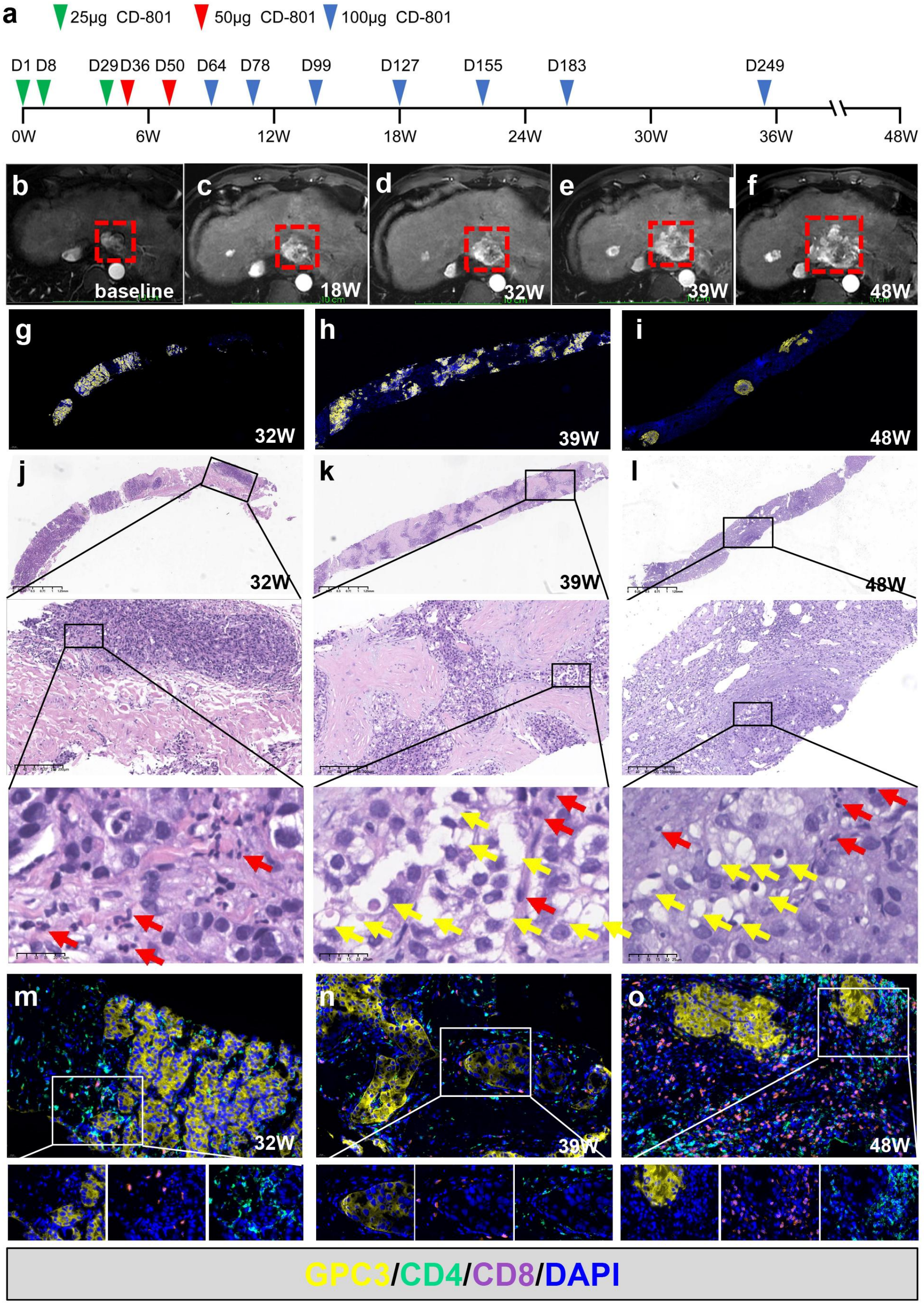


**Figure S9. Responses of patients 3 to CD-801 therapy.**

**a** A schematic representation of CD-801 treatment for patient 3. **b-f** Representative contrast-enhanced MRI images of the target lesion in patient 3. The baseline lesion is outlined in red dashed line (**b**). The target lesion remained largely stable until 18

weeks after initial therapy (**c**), after which it began to gradually enlarge, coinciding with a progressive expansion of the area with diminished enhancement during the arterial phase (**d-f**). **g-i** Immunofluorescence staining of liver cancer biopsy sections revealed a progressive decrease in GPC-3 positive tumor cells (yellow), with only a few scattered foci of residual cancer nests observed at the 48-week post-treatment. **j-l** H&E staining images of liver cancer biopsy sections. Collagen tissues progressively advanced their infiltration, intertwining with the residual tumor nests leading to the disintegration of their characteristic nest-like configuration. Significant inflammatory cell infiltration (red arrows) (**j-l**, bottom) and a pronounced degeneration of the tumor cells (yellow arrows) (**k, l**, bottom) were observed within the residual tumor nests. **m-o** Representative 4-color multiplex immunofluorescence demonstrated a progressive increment in the infiltration of $CD4^+$ (green) and $CD8^+$ (purple) T cells within the tumor tissue after the treatment.

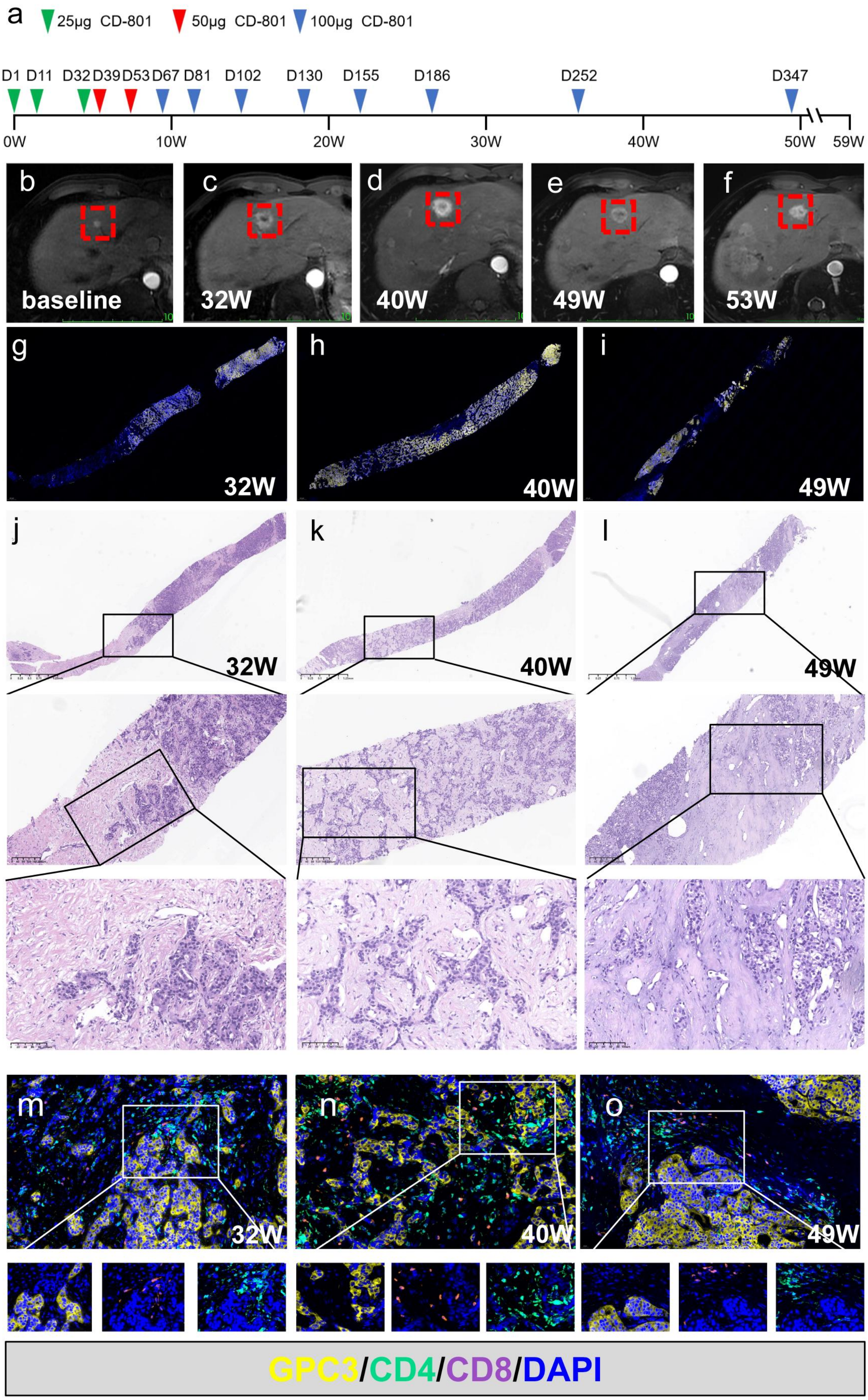
a
25μg CD-801
50μg CD-801
100μg CD-801
D1 D11 D32 D39 D53 D67 D81 D102 D130 D155 D186 D252 D347
0W 10W 20W 30W 40W 50W 59W
b baseline
c 32W
d 40W
e 49W
f 53W
g 32W
h 40W
i 49W
j 32W
k 40W
l 49W
m 32W
n 40W
o 49W
GPC3/CD4/CD8/DAPI

**Figure S10. Responses of patient 2 to CD-801 therapy.**

**a** A schematic representation of CD-801 treatment for patient 2. **b-f** Representative contrast-enhanced MRI images of one tumor lesion in patient 2. The lesion in the left medial lobe of the liver at the baseline is outlined with a red dashed line (**b**). The lesion had increased in size compared to baseline at 32 weeks post-treatment (**c**), but it remained stable from then until week 53, with a decline in arterial phase enhancement at the center during treatment (**c-f**). **g-i** Immunofluorescence staining of liver cancer tissues from biopsy showed a progressive reduction of GPC-3 positive tumor cells (yellow) following CD-801 treatment. **j-l** H&E staining images of liver cancer biopsies. Microscopic examination revealed a large amount of fibrous tissue which infiltrated the residual cancer nests at 32 weeks post-treatment (**j**). By 40 weeks, collagen progressively infiltrated and became interwoven with residual tumor nests, resulting in the disintegration of their quintessential nest-like structure (**k**). By 49 weeks, the tumor tissue was characterized by a predominant collagen deposition, with the remaining tumor cells exhibiting marked signs of degeneration (**l**). **m-o** Representative 4-color multiplex immunofluorescence revealed moderate infiltration of $CD4^+$ (green) and $CD8^+$ (purple) T cells within the tumor tissue.

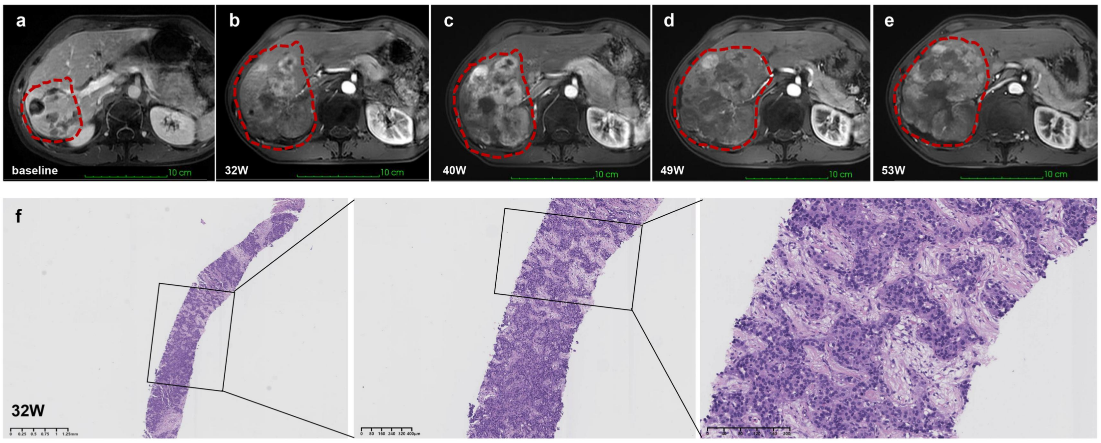


**Figure S11. Responses of patient 2 to CD-801 therapy.**

**a-e** Representative contrast-enhanced MRI images of one tumor lesion in the right posterior lobe of the liver in patient 2. The lesion is outlined with a red dashed line. The lesion had increased in size compared to baseline at 32 weeks post-treatment, but it remained stable from then until week 53, with a decline in arterial phase enhancement at the center during the treatment. **f** H&E staining images of liver cancer biopsies. Microscopic examination revealed excessive collagen deposition which infiltrated the residual cancer nests at 32 weeks post-treatment.

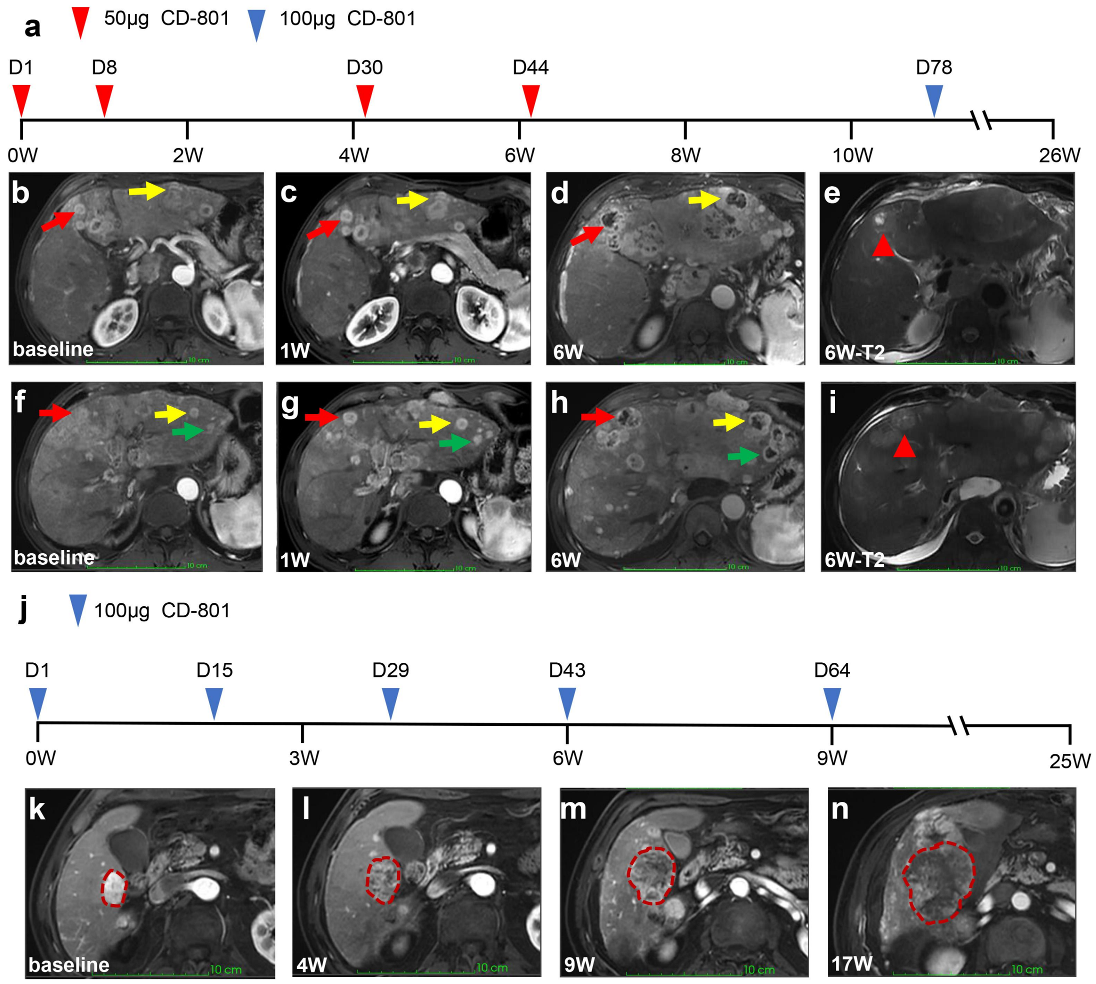


**Figure S12. Responses of patient 5 and patient 9 to CD-801 therapy.**

**a-i** Responses of patient 5 to CD-801 therapy. **a** A schematic representation of CD-801 treatment for patient 5. **b-e** and **f-i** Representative contrast-enhanced MRI images of the lesions at different anatomical planes in patient 5 following CD-801 treatment. MRI indicated that the lesions were slightly enlarged at 1 week post-treatment (comparing c to b and g to f) and revealed numerous necrotic cavities of diverse sizes within the tumors (arrows), partly encircled by a thin, uniform peripheral enhancement at 6 weeks post-treatment (comparing d to b and h to f). The same-colored arrow indicated the corresponding lesion. T2-weighted images (T2WI) showed corresponding hyperintense lesions in some of necrotic cavities (**e, i**). The red

arrowheads in figures e and i corresponded to the same lesion indicated by the red arrow in figures d and h, respectively. **j-n** Responses of patient 9 to CD-801 therapy. **j** A schematic representation of CD-801 treatment for patient 9. **k-n** Representative contrast-enhanced MRI images of the target lesion (outlined in red dashed line) in patient 9. The target lesion at baseline shows significant enhancement in the arterial phase at baseline (**k**) and was gradually enlarged accompanied by reduced enhancement in the arterial phase during treatment (**i-n**). The enhancement within the entire lesion had largely faded by 17 weeks (**n**).

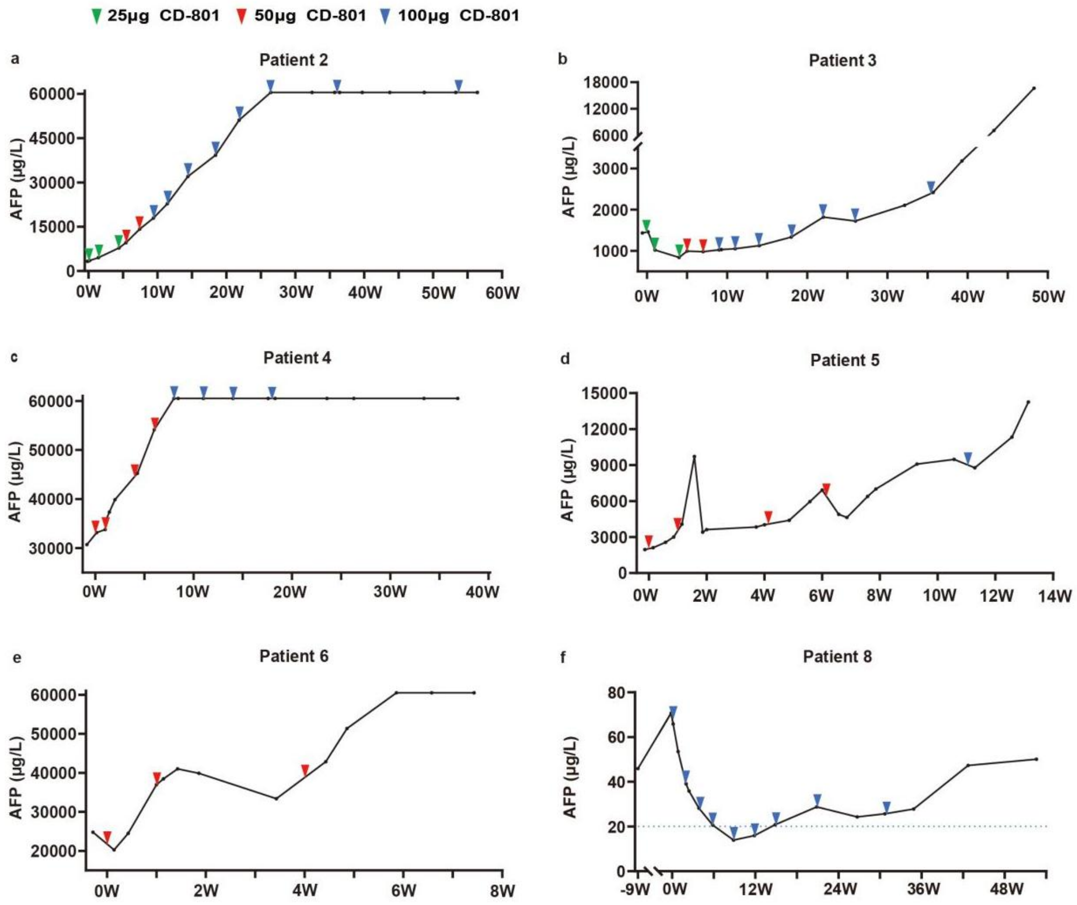


**Figure S13. Changes in serum AFP levels after CD-801 treatment.**

**a-e** Five patients with elevated serum AFP levels before enrollment showed a rapid or fluctuating increase in AFP levels after CD-801 treatment. **f** In patient 8, AFP levels declined to within the normal range (0 - 20 ng/mL) after 6 weeks of treatment and remained marginally above the range thereafter.

**Table S1. Baseline demographic and clinical characteristics of the enrolled patients.**

| **Baseline clinical characteristics** | |
|---|---|
| Age, years | |
| Median (IQR) | 57（19.5） |
| Range | 43-74 |
| Gender (male/female) | 9/0 |
| Race, Ethnicity | |
| Asian, Han Chinese, n | 9 |
| Body weight, kg | |
| Median (IQR) | 60.2（11.55） |
| < 60kg / ≥ 60 kg, n | 4/5 |
| BMI, $kg/m^2$ | |
| Median (IQR) | 22.3（5.15） |
| < 23 $kg/m^2$/≥ 23 $kg/m^2$, n | 5/4 |
| ECOG PS | |
| 0/1/2 | 6/2/1 |
| Etiology | |
| HBV | 9 |
| Alcoholic | 2 |
| Evidence of cirrhosis | |
| Yes/No | 7/2 |

| | |
|---|---|
| Child–Pugh score | |
| 5 | 3 |
| 6 | 3 |
| 7 | 2 |
| 8 | 1 |
| AFP, μg/L | |
| < 400 μg/L /≥ 400 μg/L, n | 2/7 |
| Extrahepatic spread, n | |
| Yes/No | 5/4 |
| Vascular invasion, n | |
| Yes/No | 3/6 |
| CNLC stage, n | |
| II/III | 3/6 |
| BCLC stage, n | |
| B/C | 3/6 |
| Previous treatments, n | |
| Surgical resection | 2 |
| Local ablative therapy [a] | 3 |
| Transarterial therapy [b] | 8 |
| Radiotherapy | 1 |
| Systemic therapy [c] | 7 |

IQR, interquartile range; BMI, Body Mass Index; ECOG PS, Eastern Cooperative

Oncology Group performance status; HBV, Hepatitis B Virus; AFP, α-fetoprotein; CNLC, China Liver Cancer; BCLC, Barcelona Clinic Liver Cancer. [a] Local ablative therapy includes radio frequency ablation and microwave ablation. [b] Transarterial therapy includes transcatheter arterial chemoembolization and hepatic artery infusion chemotherapy. [c] Systemic therapy includes antiangiogenic targeted therapies, immunotherapy and chemotherapy.

**Table S2. Adverse events reported during the study**

| AEs | No. of patients with any grade AEs | | |
|---|---|---|---|
| | Possible related | Not related | Total |
| **Symptomatic AEs** | | | |
| Fever | 6 | 2 | 7 |
| Pain | 7 | 4 | 8 |
| Abdominal distension | 4 | 3 | 7 |
| Anorexia | 3 | 3 | 4 |
| Edema limbs | 2 | 2 | 4 |
| Malaise | 3 | 2 | 4 |
| Hiccups | 2 | ·· | 2 |
| Diarrhea | 2 | ·· | 2 |
| Tumor hemorrhage | 2 | ·· | 2 |
| Cough | 1 | 3 | 3 |
| Ascites | 2 | 2 | 4 |
| Vomiting | 1 | 1 | 2 |
| Nausea | 1 | 1 | 2 |
| Pleural effusion | 1 | ·· | 1 |
| Fatigue | 2 | ·· | 2 |
| Infusion related reaction | 1 | ·· | 1 |
| Cytokine release syndrome | 1 | ·· | 1 |
| Upper gastrointestinal | ·· | 1 | 1 |

| | | | |
|---|---|---|---|
| hemorrhage | | | |
| Esophageal varices | ·· | 1 | 1 |
| Tinnitus | ·· | 1 | 1 |
| Pruritus | ·· | 1 | 1 |
| Eczema | ·· | 1 | 1 |
| Enterocolitis infectious | ·· | 1 | 1 |
| Upper respiratory infection | ·· | 2 | 2 |
| Otitis externa | ·· | 1 | 1 |
| Choledocholithiasis | ·· | 1 | 1 |
| Nail infection | ·· | 1 | 1 |
| Weight loss | | 1 | 1 |
| Ileus | | 1 | 1 |
| **Laboratory AEs** | | | |
| Hypoglycemia | 6 | ·· | 6 |
| Hepatitis B reactivation | 6 | ·· | 6 |
| Hepatobiliary disorders | | | |
| ALT increased | 4 | 1 | 5 |
| AST increased | 4 | 1 | 5 |
| AKP increased | 4 | ·· | 4 |
| TB increased | 3 | 1 | 4 |
| LDH increased | 3 | ·· | 3 |
| GGT increased | 2 | ·· | 2 |

| | | | |
|---|---|---|---|
| Coagulation disorder | | | |
| INR increased | 4 | 1 | 5 |
| APTT prolonged | 1 | ·· | 1 |
| Hypoalbuminemia | 2 | 1 | 3 |
| Anemia | 1 | 2 | 3 |
| Hyponatremia | 1 | 3 | 4 |
| Cholesterol high | ·· | 2 | 2 |
| Creatine phosphokinase increased | ·· | 1 | 1 |
| Hyperglycemia | ·· | 2 | 2 |
| Glucosuria | ·· | 1 | 1 |
| Hyperuricemia | ·· | 1 | 1 |
| Platelet count decreased | ·· | 1 | 1 |

| **Grade 3 or 4 AEs** | **No. of patients** | **patient** | **Relationship to treatment** |
|---|---|---|---|
| AST increased | 4 | patient 4, 5, 7, 9 | Possible |
| GGT increased | 2 | patient 4, 9 | Possible |
| TB increased | 1 | patient 4 | Possible |
| Malaise | 1 | patient 4 | Possible |
| Ascites | 1 | patient 4 | Possible |
| ALT increased | 1 | patient 5 | Possible |
| Pain | 1 | patient 9 | Possible |

| | | | |
|---|---|---|---|
| Tumor hemorrhage | 1 | patient 9 | Possible |
| Diarrhea | 1 | patient 7 | Possible |
| Ileus | 1 | patient 3 | Possible |
| Choledocholithiasis | 1 | patient 2 | Not related |
| Upper gastrointestinal hemorrhage | 1 | patient 6 | Not related |
| Cholesterol high | 1 | patient 7 | Not related |
| Enterocolitis infectious | 1 | patient 9 | Not related |

AEs, adverse events; AST, aspartate aminotransferase; AKP, alkaline phosphatase; ALT, alanine aminotransferase; TB, total bilirubin; LDH, lactate dehydrogenase; GGT, Gamma-glutamyltransferase; INR, international normalized ratio; APTT, activated partial thromboplastin time.

**Table S3. Serious adverse events reported during the study**

| SAEs | No. of patients | patient | Relationship to treatment |
|---|---|---|---|
| TB increased | 1 | Patient 4 | Possible |
| Ascites | 1 | Patient 4 | Possible |
| Cytokine release syndrome | 1 | Patient 5 | Possible |
| Tumor hemorrhage | 1 | Patient 9 | Possible |
| Ileus | 1 | Patient 3 | Possible |
| Choledocholithiasis | 1 | Patient 2 | Not related |
| Upper gastrointestinal hemorrhage | 1 | Patient 6 | Not related |
| Enterocolitis infectious | 1 | Patient 9 | Not related |

SAEs, severe adverse events; TB, total bilirubin.

**Table S4. The treatment dosage and the death causes of the deceased patients by the cutoff date.**

| Patient | Dosage（μg） | Death causes | Days post-last treatment |
|---|---|---|---|
| Patient 1 | 25; 25 | Spontaneous bacterial peritonitis and metastatic peritoneal cancer | 39 |
| Patient 4 | 50; 50; 50; 50; 100; 100; 100; 100 | Liver failure | 155 |
| Patient 5 | 50; 50; 50; 50; 100 | Severe Herpes-zoster virus infection and drug-induced liver injury | 105 |
| Patient 6 | 50; 50; 50 | Tumor progression | 65 |
| Patient 7 | 100;100;100;100;100 | Tumor progression and pulmonary metastases | 47 |
| Patient 9 | 100;100;100;100;100 | Respiratory failure | 111 |

**Table S5. Primers for Real-time RT-PCR**

| Gene | Forward primer (5'- 3') | Reverse primer (5'- 3') |
|---|---|---|
| *ACTB* (H) | CATCCTGCGTCTGGACCT | GTACTTGCGCTCAGGAGGAG |
| *Actb* (m) | GTGACGTTGACATCCGTAAAGA | GCCGGACTCATCGTACTCC |
| *ALDOB* | AGGAGGACTCTTCTCTCCCAA | GATTCATCTGCAGCCAGGAT |
| *G6PD* | GGCTCCATGACTGTGGGATC | TTCAGCTGCACAGCCCAGAA |
| *GYS2* | CCAGTGGGAAGTCGAAGAAC | TTCTCTCCCCATTCATCTGC |
| *PEPCK* | GTGTCCCTCTAGTCTATGAAGC | ATTGACTTGATCCTCCAGATAC |
| *APOC3* | GGGTACTCCTTGTTGTTGC | AAATCCCAGAACTCAGAGAAC |
| *TTR* | TCAGAAAGGCTGCTGATGAC | AGTCGTTGGCTGTGAATACC |
| *BR* | ACAAGGTGCTGCGGGAATCA | ACTGGTGGGAGGGGTAGGTG |
| *LIN28* | TGTAAGTGGTTCAACGTGCG | CCTCACCCTCCTTCAAGCTC |

| | | |
|---|---|---|
| *SMO* | ATCTCCACAGGAGAGACTGGTTCGG | AAAGTGGGGCCTTGGGAACATG |
| *MYC* | TGGAAAACCAGCCTCCCG | TTCTCCTCCTCGTCGCAGTA |
| *ESG1* | GGCGCAGTATCACAGCCTTA | GAGGTAGACCTGGTAGGCCA |
| *BMI1* | TGGCTCGCATTCATTTTCTGC | GAACAATACACGTTTTACAGAAGGA |
| *POU5F1* | CGACCATCTGCCGCTTTGAG | CCCCCTGTCCCCCATTCCTA |
| *HNF4A* | AGAAGGCACCAACCTCAACGC | CCACTCAACGAGAACCAGCAG |
| *HNF4A* (srRNA) | TCTAGTCCGCCAAGATGCGA | ATTCCAGGGTGGTGTAGGCT |

*ACTB,* Actin beta*;* *ALDOB*, aldolase B; *G6PD*, glucose-6-phosphatase; *GYS2*, glycogen synthetase 2; *PEPCK*, phosphoenolpyruvate carboxykinase; *APOC3*, apolipoprotein C 3; *TTR*, transthyretin; *BR*, biliverdin reductase; *LIN28*, lin-28 homolog A; *SMO*, smoothened, frizzled class receptor; *MYC*, MYC proto-oncogene, bHLH transcription factor; *ESG1*, embryonic cell-specific gene 1; *BMI1*, BMI1 proto-oncogene, polycomb ring finger; *POU5F1*, POU class 5 homeobox 1; *HNF4A*, hepatocyte nuclear factors-4α; *HNF4A* (srRNA), special primer for detecting srRNA-expressed HNF4α.

**Table S6. Definition of dose-limiting toxicities**

| Toxicity Category | Toxicity CTCAE Grade |
|---|---|
| Hematologic | Grade 3 or higher febrile neutropenia |
| | Grade 4 or higher neutropenia lasting ≥ 7 days |
| | Grade 4 or higher thrombocytopenia lasting ≥ 7 days or Grade 3 or higher thrombocytopenia associated with clinically significant bleeding |
| | Any other Grade 4 or higher hematological toxic effects |
| Non-hematologic | Grade 4 or higher non-hematological toxicities |
| | Grade 3 non-hematological toxicities lasting > 3 days (excluding nausea, vomiting, diarrhea, electrolyte imbalance, and rash controlled by medical intervention within 72 hours) |
| | Grade 3 CRS persisting for ≥ 72 hours despite optimal supportive treatment, or Grade 4 CRS |
| | Any Grade 3 or higher non-hematologic laboratory value requiring treatment (except AST/ALT) |
| | AST/ALT > 20.0 ULN if the baseline was normal, or > 20.0 times the baseline if the baseline was abnormal |
| | Bilirubin > 10.0 ULN if the baseline was normal, or > 10.0 times the baseline if the baseline was abnormal, which does not resolve within 1 week or is clinically symptomatic |

CTCAE, Common Terminology Criteria for Adverse Events; CRS, Cytokine Release

Syndrome; AST, aspartate aminotransferase; ALT, alanine aminotransferase; ULN, the upper limit of normal.

**SUPPLEMENTARY MATERIALS AND METHODS**

## Investigational agent CD-801

In this study, CD-801, a lipid nanoparticle (LNP)-encapsulated self-replicating RNA (srRNA) encoding HNF4α, was developed using methods as described previously.[1, 2] The srRNA was engineered using an alphavirus genomic backbone, retaining non-structural protein genes (nsp1-4) for RNA replication while replacing structural proteins with the HNF4α coding sequence. The construct features a 5' untranslated region (UTR), 26S subgenomic promoter, and 3' polyadenylated tail. LNPs were formulated via microfluidic mixing (Micro&Nano) of an aqueous phase containing srRNA in 50 mM citrate buffer (pH 6) with an ethanol phase containing 1,2-Diastearoyl-sn-glycero-3-phosphocholine (DSPC), Cholesterol, 1,2-dimyristoyl-sn-glycero-3-phosphoethanolamine-N-[methoxy (polyethylene glycol)-2000] (DMG-PEG 2000) and DHA-1 (Sinopeg, 06040009300) at a 9.4:42.5:1.8:46.3 molar ratio (Nitrogen of ionizable lipid: phosphate of mRNA ratio 6). Quality testing by Hangzhou Immona Medical Health Technology Co., Ltd. (Batch No. 202305002) confirmed compliance with specifications (IMMO-801-STD.08.003/01). The formulation appears as a colorless opalescent suspension (pH 7.2) with osmolality 318 mOsmol/kg and minimal subvisible particles (34 particles ≥10 μm/vial, 0 particles ≥25 μm/vial). Nanoparticles demonstrated optimal characteristics: 56 nm diameter, 0.198 polydispersity index, and -12.87 mV zeta potential. The product contains 50.6 μg/mL RNA with 91.3% encapsulation efficiency, validated sequence accuracy, and confirmed HNF4α expression in vitro.

Packaged as 25 μg/0.5 mL vials, CD-801 remains stable when stored light-protected at ≤ -60°C (Supplementary Data S1). When removing from the freezer, allow the vial to thaw at room temperature for approximately 20 minutes. After thawing, gently agitate the vial to ensure homogeneity, avoiding the use of a vortex mixer or inverting the vial. It is imperative that the injection be completed within 4 hours following the thawing of the investigational agent CD-801. Light protection is not necessary for the subsequent steps of dilution and administration. Additionally, an LNP-encapsulated green fluorescent protein srRNA (*GFP* srRNA) was established as a control for *in vitr*o study, whereas the structural protein sequences were replaced with the gene sequence of *GFP*.

## **Preclinical study**

### **Effect of CD-801 on the hepatocellular carcinoma (HCC) cells *in vitro***

#### ***Cell lines and cell cultures***

The human HCC cell line Huh-7 was obtained from the Type Culture Collection of the Chinese Academy of Sciences (Shanghai, China) and were cultured in Dulbecco's modified Eagle's medium (DMEM) supplemented with 10% heat-inactivated fetal bovine serum (FBS). The primary human hepatocytes (PHH) were obtained from LV BioTech（Shenzhen）and were cultured following the manufacturer's instructions.

#### ***srRNA delivery***

Huh-7 cells were seeded at a density of $3\times10^5$ cells per well in a 6-well plate and were cultured at 37°C overnight. Cells were washed with PBS and were added with indicated doses of srRNA diluted in 1 mL Opti-MEM (Thermo fisher). The same

volume of DMEM containing 20% FBS was added to the cells 6 hours after the transfection. To evaluate the transfection efficiency, *GFP* srRNA transfected cells were detached with trypsin, and collected in PBS solution at 24 hours post transfection. GFP expression was evaluated by flow cytometry (Attune NxT, Invitrogen, China). The percentages of GFP-positive cells were quantified and analyzed by Flowjo software. To detect the expression of target genes, the transfected cells were collected for Western blotting and RT-PCR at 1, 3, 5 or 7 days post transfection.

***Cell proliferation assay***

Huh-7 cells were seeded at a density of $5\times10^3$ cells per well in a 96-well plate. After transfection with the corresponding concentration of CD-801 or *GFP* srRNA, metabolically active cells were assessed using the Cell Counting Kit-8 (CCK8, Dojindo, Tokyo, Japan) daily for 5 days.

***Colony Formation Ability Assay***

Huh-7 cells were plated with a density of $3\times10^3$ cells per well in a 96-well plate treated with CD-801 or *GFP* srRNA at 1μg / $3\times10^5$ cells for 24 hours. The cells were detached with trypsin and transfer to 6 well plate. Following a 2-week incubation, the plates were washed with PBS and fixed in 4% paraformaldehyde for 20 min and then stained with 0.005% crystal violet. The images were scanned and analyzed.

***Periodic acid-Schiff reaction***

To evaluate the stored glycogen, Huh-7 cells were seeded at a density of $3\times10^4$ cells per well in a 24-well plate and treated with CD-801 or *GFP* srRNA at a density of 1

μg / $3\times10^5$ cells. After 3 days, cells were stained with a periodic acid-Schiff (PAS) reaction kit (Beyotime, China) according to the manufacturer's protocol. Briefly, the cells were fixed in 75% ethanol for 15 minutes and subsequently oxidized in periodic acid for 10 minutes. The cells were submerged in Schiff's reagent for 40-60 minutes after PBS washing, and then stained with 100 μl of hematoxylin staining solution for 30 seconds. Glycogen storage was visualized under a light microscope. Image analysis software (Image-Pro Plus 6.0, Media Cybernetics) was used to quantify the staining area.

***Acetylated low-density lipoprotein uptake assay***

The acetylated low-density lipoprotein (ac-LDL) uptake assay was performed to evaluate the metabolic function. Huh-7 cells were plated at a density of $3\times10^4$ cells per well in 24-well plates and treated with 0.1 μg CD-801 or *GFP* srRNA for 3 days. The medium was then replaced by fresh medium containing Dil-Ac-LDL (Invitrogen) at a ratio of 1:100 for 4 h at 37°C. Each well was then imaged using fluorescence microscopy. Image analysis software (Image-Pro Plus 6.0, Media Cybernetics) was used to quantify the ac-LDL positive area.

***Senescence associated β-galactosidase activity assay***

The β-galactosidase (β-gal) activity assay was performed to assess the cell senescence. Huh-7 cells were seeded at a density of $3\times10^5$ cells per well in 6-well plates and treated with 1.0 μg CD-801 or *GFP* srRNA for 3 days. Cell senescence of the HCC cells was assessed using a senescence β-gal staining kit (Beyotime, China). After fixing with 4% paraformaldehyde for 15 minutes, the cells were incubated with fresh

senescence-associated β-gal staining solution at 37 ℃ overnight. Senescent cells were visualized and captured under a microscope. Image analysis software (Image-Pro Plus 6.0, Media Cybernetics) was used to quantify the senescent cell area.

***Apoptosis assay***

Huh-7 cells were seeded at a density of $3\times10^5$ cells per in in a 6-well plate and then were treated with 1.0 μg CD-801 or *GFP* srRNA for 3 days. Apoptotic cells were detected using an APC Annexin V/PI Apoptosis Kit (Biolegend, China) according to the manufacturer's instructions. The cells were harvested using 0.25% trypsin, washed twice with PBS, and transferred to a test tube, where they were resuspended in 100 μl of Binding Buffer. To each tube, 400 μl of Binding Buffer containing 5 μl of APC Annexin V and 5 μl of propidium iodide were added, and the cells were incubated for 15 minutes at 25 ℃ in the dark. Subsequently, the cells were analyzed using flow cytometry (Attune NxT, Invitrogen, China). Flow cytometric data were analyzed using FlowJo V10 software.

***Human albumin ELISA***

Huh-7 cells were seeded at a density of $3\times10^5$ cells per well in 6-well plate and treated with 1.0 μg CD-801 or *GFP* srRNA for 24 hours. The cell culture media was centrifuged at 1500 rpm for 10 minutes at 4 ℃ to remove debris. Human Albumin was measured by the Human Albumin ELISA Quantitation kit (Assay Pro) according to the manufacturer’s instructions. The value was normalized to the corresponding cell counts of each group to calculate albumin secretion per $10^5$ cells.

***Urea secretion assay***

Huh-7 cells were plated at a density of $3\times10^5$ cells per well in 6-well plate and treated with 1.0 μg CD-801 or *GFP* srRNA for 2 days. Urea secretion in the supernatant was measured using the QuantiChromTM Urea Assay Kit (BioAssay System). Urea secretion levels were normalized to the cell counts of each group.

***Real-time RT-PCR***

Total RNA was extracted from cells or tissues following standard Trizol (Takara) protocol. The cDNA synthesis for cells was carried out using the RT Master Mix (Takara) with the isolated total RNA. For tissue samples, the cDNA synthesis was conducted using M-MLV Reverse Transcriptase (Promega). Transcript levels were quantified using SYBR Green-based real-time PCR with the ABI StepOne Real-time PCR Detection System (Life Technologies). The analysis of gene expression was conducted using the $2^{-\Delta\Delta CT}$ method. Each condition was assessed in at least three independent experiments. The primer sequences are provided in Table S5. The primer *HNF4α* (srRNA), specifically designed to target the srRNA-expressed human *HNF4α* sequence, was developed to precisely evaluate the self-amplified level of CD-801.

***ELISA assay***

The levels of human HNF4α in nude mouse organs and xenograft tumor tissue extracts were measured by ELISA kit (Abcam, ab210581) following the manufacturer's instructions.

***Western blotting analysis***

Cells were lysed with lysis buffer (125 mM Tris-HCl pH 6.8, 25% glycerol, 5% SDS) supplemented with protease inhibitor (Beyotime, China), separated on SDS-PAGE,

and then transferred onto nitrocellulose membranes (HAHY00010, Millipore). After blocking in PBST containing 5% skim milk for 1 hour, the membranes were incubated with a primary antibody (HNF4α antibody, Sigma-Aldrich, HPA004712; β-Actin antibody, abclonal, AC038; GFP antibody, Santa Cruz, SC9996; P21 antibody, proteintech, 10355-1-AP; P16 antibody, proteintech, 10883-1-AP; γ-H2AX antibody, Abcam, ab81299) overnight at 4℃. Then the membranes were incubated with a secondary antibody (donkey-anti-mouse or donkey-anti-rabbit, IRDye 680 or IRDye 800, respectively). The signals were then subsequently quantified using an Odyssey infrared imaging system (LI-COR) at 700 nm or 800 nm.

**Anti-tumor effect of CD-801 on human HCC xenograft models**

Male athymic BALB/c nude mice were purchased from Shanghai BK/KY Biotechnology company (Shanghai, China) and housed under specific pathogen-free conditions with a 12-hour on/off light cycle. Tumor xenografts were generated by subcutaneously injecting Huh-7 cells ($1\times10^6$) into the right flank of nude mice (5 weeks old). Upon reaching an average volume of approximately 150 $mm^3$, the mice were randomly divided into three groups (seven animals per group). Intratumoral injections of normal saline or 5 µg of *GFP* srRNA or CD-801 in a final volume of 75 µl were administrated. Tumor volume was measured daily post-injection and calculated using the formula: volume = length × $(width)^2$ × 1/2. After five days post-injection, the mice were sacrificed, and tumors were excised and weighed.

For the orthotopic HCC model, the luciferase-expressing Huh-7 cells ($1\times10^6$) were initially injected subcutaneously into the right flank of BALB/c nude mice to generate

tumor xenografts. The tumor nodules from the subcutaneous xenograft model were cut into 1 $mm^3$ pieces and implanted into the left lobe of the livers of BALB/c nude mice (male, 7 weeks old). For in vivo observation of tumor size, the tumor-bearing mice were intraperitoneally injected with D-luciferin potassium salt at a dosage of 150 mg/kg and then imaged through the Caliper IVIS Lumina bioluminescence imaging system (PerkinElmer, Waltham, MA, USA) 5 minutes later. To determine the distribution of CD-801 expression across various organs and tumor tissues, Huh-7 orthotopic tumor-bearing mice were injected via the tail vein with a single dose (2 mg/kg) of either CD-801 or *GFP* srRNA. Two or three mice per group were euthanized, and different organs and tumor tissues were collected at multiple time points post-treatment. The RNA levels of human *HNF4α*-driven by srRNA in different organs and tumor tissues were detect by RT-PCR. The protein level of HNF4α in tumor tissues were then assessed through ELISA, western blotting and immunohistochemical (IHC) analysis. The quantification of hepatocyte-specific markers was conducted using RT-PCR.

To determine the anti-tumor effect of CD-801, the Huh-7 orthotopic tumor-bearing mice were randomly divided into three groups (eight animals per group) according to the bioluminescence 3 days after implantation and then were administrated with two doses of normal saline, *GFP* srRNA, or CD-801 at seven-day intervals via the tail vein injection at the nanoparticles dose of 2 mg/ kg in a final volume of 200 µl. The tumors were monitored using bioluminescence imaging at 15 days post-treatment. Mice were sacrificed, and tumors were excised and weighed at 17 days post-treatment.

Tumor samples from these in vivo studies were collected for further Western blotting and histopathological examination.

***Immunohistochemistry***

For histopathological evaluation, formalin-fixed and paraffin-embedded sections of tumor tissues were utilized. Standard staining with hematoxylin and eosin or immunohistochemistry analysis was conducted on 4-mm thick sections from each specimen block and the prepared slides were then observed under an Olympus photomicroscope in Tokyo, Japan. The following primary antibodies were used: rabbit anti-HNF4α (Abcam, ab181604), and mouse anti-Ki67 (Serverbio, GB11149). The intensity of positive staining was measured by Image-Pro Plus 6.0 (Media Cybernetics) image analysis software.

***Immunofluorescence staining***

The prepared tissue slides were deparaffinized and rehydrated using xylene and graded ethanol. Following incubation with antigen retrieval in Tris-EDTA buffer, the tissues were blocked with 5% BSA. Primary antibodies were incubated at 4°C overnight, followed by incubation with Alexa Fluor 555- and Alexa Fluor 647-conjugated secondary antibodies (Thermo Fisher, Waltham, MA, USA). The slides were mounted with DAPI-containing medium (Sigma-Aldrich, DUO82040), and the images were captured and scanned using K-viewer software from KFBIO technology. The primary antibodies included: Anti-Ki67 (Serverbio, GB121141), Anti-HNF4α (Thermo Fisher, MA5-14891).

**Assessment of the acute toxicity**

In this single-dose toxicity study, Sprague-Dawley (SD) rats were administrated a single dose of 150 μg CD-801 via intravenous injection with a 14-day observation period. LNP was used as the vehicle control group to evaluate the toxicity of the delivery system, while 0.9% sodium chloride injection served as the negative control group. The dose volume of all groups was 3.0 mL per animal. Each group consisted of a total of 10 animals, with an equal distribution of male and female rats. Daily observations were made for mortality/moribundity and cage-side assessments, and the animals were weighed once a week after injection. Prior to sample collection, all animals underwent an overnight fast. Samples were collected for hematology and coagulation on Day 15, clinical chemistry on Days 4 and 15, and high-sensitivity cardiac troponin I analysis on Days 4 and 15. Necropsies were scheduled at the end of the observation period on Day 15.

## Clinical study

### Study design

This single-center, open-label, dose-escalation study to investigate the safety, tolerability and efficacy of CD-801 recruited patients with advanced HCC in Shanghai Changzheng Hospital between June 30, 2023, and August 28, 2023. The protocol was performed in accordance with the Declaration of Helsinki and approved by the institutional ethics committee (No. 2023SL036) of Shanghai Changzheng Hospital and registered on https://www.chictr.org.cn (ChiCTR2300073093). Each patient provided written informed consent. Shanghai Cell Diff Medicine Technology Limited Company (Shanghai, China) provided CD-801 and financial support for the

trial. All the authors attest to the accuracy and completeness of the data reported and adherence of the trial to the protocol. Final follow-up occurred on August 20, 2024.

## Patients

### *Inclusion criteria*

Patients were required to meet all the following criteria to be enrolled into this study:

1. Males or females, aged 18 years or older.
2. Confirmed diagnosis of HCC with any of the following criteria according to the American Association for the Study of Liver Diseases criteria：
   a) For those at highest risk with HBV/HCV infection or cirrhosis from any etiology, the diagnosis of HCC in lesions ≥ 1 cm in size could be based on noninvasive imaging criteria: arterial phase hyperenhancement (APHE) and washout on portal venous or delayed phases of contrast-enhanced multiphase CT or MRI.
   b) Histologically or cytologically confirmed diagnosis of HCC.
3. Unresectable HCC.
4. Those who are ineligible for locoregional or systemic therapies, or those who have experienced disease progression or no longer benefit from at least one of the therapies, according to any of the following criteria:
   a) CT/MRI examination showed residual tumors 1 month after 2 or more ablation treatments.
   b) After 2 or more transcatheter arterial chemoembolization (TACE) treatments, CT/MRI examination showed more than 50% definite viable disease,

development of new HCC, new vascular invasion or extrahepatic metastases, or lacked improvement for tumor markers.

c) According to mRECIST, subjects experienced disease progression while undergoing at least one systemic therapy, or were intolerant to systemic therapies.

5. According to mRECIST, subjects should have at least 1 measurable target lesion meeting the following criteria:

a) The lesion could be accurately measured in at least 1 dimension as 1 cm or more.

b) The lesion is suitable for repeat measurement.

c) The lesion shows intratumoral arterial enhancement on contrast-enhanced MRI.

Lesions previously treated with surgical resection, radiotherapy, or locoregional therapy must show radiographic evidence of disease progression according to mRECIST to be deemed a target lesion.

6. Life expectancy of 12 weeks or more.

7. Subjects must have an Eastern Cooperative Oncology Group (ECOG) Performance Status (PS) of 0 to 2.

8. Males with fertility and females of childbearing potential are willing to use a highly effective method of contraception for the entire study period and for 6 months after study drug discontinuation. Females of childbearing age, including premenopausal females and within 2 years after menopause, must have a negative

serum pregnancy test result within 7 days prior to the first dose of study treatment.

9. Subjects who had a voluntary agreement to provide written informed consent and the willingness and ability to comply with all aspects of the protocol.

***Exclusion criteria***

Patients with any of the following criteria were excluded from participation in this study:

1. Inadequate liver function: Albumin (ALB) < 28 g/L, or total bilirubin > 5·0 mg/dL, or aspartate aminotransferase (AST), alkaline phosphatase (ALP), or alanine aminotransferase (ALT) > 5 × the upper limit of normal (ULN).
2. Inadequate renal function defined as creatinine > 1.5 × ULN or calculated creatinine clearance < 40 mL/min.
3. Absolute neutrophil count (ANC) < $1.0\times10^9$/L, or Platelets < $30\times10^9$/L, or Hemoglobin < 8.5 g/dL.
4. International normalized ratio (INR) > 2.3.
5. History of liver transplantation.
6. Poorly controlled hypertension, diabetes or other serious heart or lung diseases, or with serious dysfunction.
7. Extrahepatic metastasis who had not received first-line systemic therapies (excluding those who are not eligible for systemic therapies) or who were receiving effective systemic therapy currently.
8. History of anticancer treatment with any locoregional therapies, antiangiogenic

targeted therapies, immune checkpoint inhibitors or chemotherapy (within 4 weeks, or within 2 weeks in case of sorafenib), radiotherapy (within 3 weeks), or active traditional Chinese medicine (within 2 weeks) before the first dose of study treatment, except for the treatments after which the disease still progressed according to mRECIST.

9. All toxicities related to prior locoregional or systemic anti-tumor treatments are still grade 2 or more (except for hair loss and other events that have been judged tolerable by researchers).
10. Complication histories of liver cirrhosis or HCC such as gastrointestinal hemorrhage, overt hepatic encephalopathy, or refractory ascites within 2 weeks prior to the first dose of study treatment.
11. Uncontrolled active infection (eg, lung infections, or abdominal infections).
12. Moderate to severe hepatic artery- portal vein fistula or hepatic artery - vein fistula which could not be avoided even through superselective catheterization was performed.
13. History of malignancy other than HCC within 5 years prior to screening, with the exception of malignancies with a negligible risk of metastasis or death (e.g., 5-year overall survival rate > 90%), such as adequately treated early gastric carcinoma, carcinoma in situ of the cervix, non-melanoma skin carcinoma, or localized prostate cancer.
14. HBV DNA greater than 500 copies/mL, or HCV RNA greater than 100 IU/ml.
15. Positive for human immunodeficiency virus (HIV).

16. Allergic to MRI contrast agents.
17. Pregnant/lactating women, or women with the possibility of pregnancy.
18. Participation in other investigational drug trials within 4 weeks prior to initiation of this study treatment.
19. Any medical conditions which, in the opinion of the investigator, would preclude participation in this clinical trial.

**Trial design and procedures**

In this dose-escalation study, the patients were sequentially allocated to receive 25 μg, 50 μg, and 100 μg of CD-801 administration. Based on the i3+3 design,[3] the subsequent patients could be allowed to be recruited into the higher dose group only if all 3 patients in the lower dose group completed their dose-limiting toxicities (DLT) observation without any observed DLT. Otherwise, another 3 participants should be enrolled in the same dose group. The re-treatment protocol was determined by the researchers and members of the expert committee based on the DLT, the adverse events in the observation period, and the efficacy. In general, upon completion of the DLT observation for participants in the higher dosage group, the lower dosage group would be discussed to decide whether to increase the dosage. The treatment was continued until disease progression, development of unacceptable toxicity, or withdrawal of consent.

Safety evaluations were conducted, encompassing a comprehensive medical history, vital sign monitoring, physical examinations, ECOG PS, and laboratory tests which included a complete blood count, blood biochemistry, coagulation panel, cardiac

enzyme profile, lipid profile, urinalysis, stool routine, complemented by electrocardiogram (ECG) assessments. A chest CT, abdominal contrast-enhanced MRI, and contrast-enhanced CT or MRI of suspected metastatic regions were performed for the tumor evaluation. The treatment decisions were based on mRECIST by an investigator assessment. Alpha-fetoprotein (AFP) levels were monitored throughout the treatment period in all patients and liver biopsies were performed in some patients. A final follow-up visit was scheduled 2 weeks post-final dose.

During the entire study period, participants were allowed to receive symptomatic and supportive therapy, such as adjustments of diuretic dosages, administration of albumin, anti-infection treatments, hepatoprotective and choleretic agents, as well as nutritional support therapy. Antiviral treatments that were ongoing before the study were continued without modification during the study period. Additionally, the use of other permitted medications was determined at the discretion of the researchers, ensuring it did not interfere with the observation of study endpoints.

**CD-801 administration**

Prior to administration, CD-801 was dissolved in sterile saline, with volumes of 10 ml for doses of 25 and 50 μg, and 20 ml for the 100 μg dose. Patients underwent hepatic arterial angiography in the Digital Subtraction Angiography room by interventional radiologists, accessed either through the left radial or right femoral artery. Superselective catheterization and angiography of the tumor-feeding branches were conducted using a coaxial microcatheter. Utilizing an infusion pump, CD-801 was administered through the microcatheter into the tumor-supplying arterial branches at a

controlled rate of 1 ml per minute.

**Outcomes**

The primary endpoints included the safety and tolerability assessed based on the incidence of dose-limiting toxicities (DLT) within 14 days after the initial drug administration, and the frequency and severity of adverse events, serious adverse events, and events leading to treatment discontinuation throughout the treatment period. DLT criteria were described in detail in Table S6 and adverse events were assessed according to Common Terminology Criteria for Adverse Events (CTCAE) Version 5.0.

The secondary endpoints were the clinical response of the tumor lesions to CD-801 treatment evaluated based on mRECIST criteria[4] by an independent imaging review. Serum alpha fetoprotein (AFP) levels were monitored throughout the study.

**Pathological examination of patients' biopsy samples**

The liver tumor tissue biopsies were performed on patients 2, 3, and 4. Formaldehyde-fixed, paraffin-embedded sections of these liver tissues were used for histopathological examination. The liver tissue sections were firstly stained with hematoxylin-eosin (H&E). Multiplex immunofluorescence staining (mIFS) was then performed to assess the infiltration of immune cells including $CD4^+$ and $CD8^+$ T cells and the expression of Glypican-3 (GPC-3), a specific marker for HCC. Firstly, the concentration and the order of the three antibodies were optimized. Automatic immunohistochemistry staining machine (Leica bond III) was used to operate the steps of dewaxing, antigen repair and staining. The antigen retrieval conditions were

ER2 antigen retrieval solution (Leica, AR9640-CN) at 100°C for 20 min. Multi-round sequential antibody incubation was carried out using primary antibodies including CD4 (Abcam, ab133616, 1:400), CD8 (Biolynx, Hangzhou, China, BX50036-C3, 1:300) and GPC-3 (Abcam, ab207080, 1:1000). After the last TSA cycle, the slides were mounted with antifade mountant containing DAPI (WiSee Biotechnology, A11008). After sealing, the slides were examined under a fluorescence microscope and the images were captured using a scanner (3DHISTECH, Pannoramic MIDI). Two independent, trained pathologists, blinded to treatment details, were assigned to assessed the biopsy specimen slides.

**Statistical Analysis**

In the preclinical study, data were analyzed with Prism 8 (GraphPad Software, La Jolla, CA, USA). The data were presented as the mean ± standard error of the mean (SEM). All *in vitro* data are representative of at least three independent experiments. Data between two groups were compared using Student's t-tests; one-way ANOVA was used for multiple groups. Statistical tests were two-tailed and a $p<0.05$ was considered statistically significant. In the clinical trial, the sample size was determined based on a dose-escalation plan with an i3+3 design, and the safety and efficacy analysis sets included all nine patients. Continuous parameters were expressed as the mean ± standard deviation (SD) or median and interquartile range (IQR) based on the normal distribution or non-normal distribution of the data, while categorical variables were expressed as numbers.

## REFERENCES FOR SUPPLEMENTARY APPENDIX


1. Wang, Z. et al. *Sci Rep* **14**, 7366 - 7381 (2024).

2. Geall, A.J. et al. *Proc Natl Acad Sci U S A* **109**, 14604 - 14609 (2012).

3. Liu, M., Wang, S., Ji, Y. *J Biopharm Stat* **30**, 294 - 304 (2020).

4. Lencioni, R., Llovet, J.M. *Semin Liver Dis* **30**, 52 - 60 (2010).

## SUPPLEMENTARY RESULTS

### Pre-clinical study

#### *Anti-HCC effect of CD-801 in vitro*

Real-time PCR revealed that Huh-7 HCC cells demonstrated more robust and sustained *HNF4α* expression compared to that observed in primary human hepatocytes in vitro (Supplementary information, Fig. S1a). Flow cytometry analysis of GFP expression in Huh-7 cells treated with *GFP* srRNA showed that transfection efficiency of srRNA was increased dose dependently in vitro (Supplementary information, Fig. S1b). The protein level of HNF4α was significantly upregulated in Huh-7 cells upon CD-801 delivery and the overexpression of HNF4α was persisted until day 7 (Supplementary information, Fig. S1c). As expected, CD-801 apparently inhibited the proliferation and colony formation in Huh-7 cells (Supplementary information, Fig. S1d-e). Real-time PCR showed that the transcription of hepatocyte-specific markers (*ALDOB, G-6-P, GYS2, PEPCK, APOCIII, TTR*) were significantly increased, whereas the expression of stemness-associated genes (*LIN28, SMO, C-MYC, ESG1, BMI1, OCT3/4*) were remarkedly reduced in CD-801 treated Huh-7 cells (Supplementary information, Fig. S1f). Moreover, CD-801 treatment enhanced the intake of ac-LDL, glycogen storage, albumin synthesis, and urea production, all important symbols of hepatic function, and induced the cellular senescence and apoptosis of Huh-7 cells (Supplementary information, Fig. S1g-l).

#### *Acute toxicity assessment of CD-801*

All experimental animals received a dose of 150 μg/animal of CD-801 or LNP liquid control and survived until the scheduled necropsies. At the end of the observation period, no test article-related adverse effects were noted in terms of general observations, body weight, and clinical pathology parameters (e.g. hematology, coagulation, and clinical chemistry). There were no abnormalities in high-sensitivity cardiac troponin I (cardiac injury biomarker). Additionally, no abnormal macroscopic or histopathological findings were detected in any of the animals. These findings indicated that all rats tolerated the intravenous administration of CD-801 at a single dose of 150 μg/animal well, with no test article-related toxic effects noted (Supplementary Data S2).

**SUPPLEMENTARY Data S1:** The Quality testing of CD-801



## 杭州依莫纳医疗健康科技有限公司

## 检 验 报 告 书

| | | | |
|---|---|---|---|
| 产品名称<br>Product Name | CD-801 注射液 | 批号<br>Batch No. | 202305002 |
| 生产商代码<br>Manufacture Code | IMMO-801 注射液 | 物料编码<br>Material Code | 009-005 |
| 规格<br>Specification | 25 μg（0.5 ml）/瓶 | 来源<br>Source | 杭州依莫纳医疗健康科技有限公司 |
| 检验依据<br>Reference | IMMO-801 注射液质量标准 STD.08.003/01 | 贮存条件<br>Storage Condition | -60℃及以下避光保存 |
| 生产日期<br>Manufacturing Date | 2023/05/20 | 有效期至<br>Expiration Date | 2024/11/19 |
| 检验日期<br>Test Date | 2023/05/21 | 报告日期<br>Report Date | 2023/06/12 |

| 检 测 项 目<br>Test items | 接 受 标 准<br>Acceptance Criteria | 检 测 结 果<br>Test Results |
|---|---|---|
| 外观 | 应为无色带乳光混悬液，无可见异物 | 无色带乳光混悬液，无可见异物 |
| 可见异物 | 应符合规定 | 符合规定 |
| pH 值 | 应为 6.8-7.8 | 7.2 |
| 不溶性微粒 | 含 10 μm 及 10 μm 以上的不溶性微粒不得过 6000 粒每瓶，含 25 μm 及 25 μm 以上的不溶性微粒不得过 600 粒每瓶 | ≥10μm：34 粒/瓶<br>≥25μm：0 粒/瓶 |
| 渗透压摩尔浓度 | 应为 240~360 mOsmol/kg | 318 mOsmol/kg |
| 装量 | 应不低于标示量 | 符合规定 |
| 粒径 | 应不高于 250 nm | 56 nm |
| PDI | 应不高于 0.300 | 0.198 |
| Zeta 电势 | 应在±15 mV 范围内 | -12.87 mV |
| 目的基因序列准确性 | 应与理论序列一致 | 与理论序列一致 |
| RNA 含量 | 应为 42.5-57.5 μg/ml | 50.6 μg/ml |
| 包裹率 | 应不低于 80.0% | 91.3% |
| 体外生物学活性 | 应表达 HNF4α | 有表达 |

**SUPPLEMENTARY Data S2:** Key excerpts of the final report for "A Single Dose Toxicity Study of CD-801 Injection in Sprague-Dawley Rats via Intravenous Injection"

益诺思
INNOSTAR

# Final Report

**Study Title:** A Single Dose Toxicity Study of CD-801 Injection in Sprague-Dawley Rats via Intravenous Injection

**Study Number:** NN23098AD01

**Study Director:** Wen Tang, M.S.

**Test Facility:** InnoStar Bio-tech Nantong Co., Ltd.

**Sponsor:** Shanghai Cell Diff Medicine Limited

**Issued Date:** Jul. 31, 2023

**Total Pages:** 181 pages (including this page)

# TRANSLATION STATEMENT

This is to certify that this final report of the study, A Single Dose Toxicity Study of CD-801 Injection in Sprague-Dawley Rats via Intravenous Injection (Study Number: NN23098AD01), is an English version translated from the final report in Chinese and this document intend to reflect all contents of the final report. Format changes from the original final report may have occurred during the translation by Huabin Zhou, InnoStar, which have no impact on the accuracy, quality, or integrity of the content or the conclusion of the study.

Translated by: Huabin Zhou, M.S.

Signature: Huabin Zhou Date: Aug. 18, 2023

Reviewed by: Wen Tang, M.S.

Signature: Wen Tang Date: Aug. 18, 2023

## GLP 遵从性声明和签字页

## GLP Compliance Statement and Signature

试验编号（Study Number）： NN23098AD01

试验名称（Study Title）： SD 大鼠静脉注射给予 CD-801 注射液单次给药毒性试验（A Single Dose Toxicity Study of CD-801 Injection in Sprague-Dawley Rats via Intravenous Injection）

除供试品和载体对照品的质量检测外，本试验其他阶段均在本试验机构完成，遵从下述GLP管理规范。本试验未出现影响试验质量和完整性的因素。

- NMPA (CFDA). 药物非临床研究质量管理规范
- US FDA 21 CFR Part 58. Good Laboratory Practice for Nonclinical Laboratory Studies.

供试品和载体对照品的质量检测在 GMP 条件下完成，检验报告（Certificates of Analysis，COAs）由委托方负责提供。

Except for the analyses of the test article and vehicle control, this study was conducted in the test facility according to the following GLP regulations. Nothing occurred to markedly affect the quality and integrity of the study.

- NMPA (CFDA). Good Laboratory Practice for Non-clinical Laboratory Studies.
- US FDA 21 CFR Part 58. Good Laboratory Practice for Nonclinical Laboratory Studies.

The analyses of the test article and vehicle control were conducted under GMP conditions. Certificates of Analysis (COAs) were provided by sponsor.

签名（Signature）： 唐稳 日期（Date）：2023 年 07 月 31 日

唐稳（Wen Tang），M.S. YYYY/ MM/ DD

专题负责人（Study Director）

# 质量保证声明

## Quality Assurance Statement

试验编号（Study Number）：NN23098AD01

试验名称（Study Title）：SD 大鼠静脉注射给予 CD-801 注射液单次给药毒性试验（A Single Dose Toxicity Study of CD-801 Injection in Sprague-Dawley Rats via Intravenous Injection）

本机构 QAU 对该试验进行了如下检查（除供试品和载体对照品的质量检测外），本总结报告如实描述试验材料和方法，准确完整地反映试验过程中产生的原始数据。

The conduct of the study was inspected by the QAU of the test facility as follows (except the analyses of the test article and vehicle control). The final report accurately and completely describes the materials and methods, and the reported results accurately and completely reflect the raw data of the study.

| 检查类型 (Inspection Type) | 检查项目 (Phases Inspected) | 检查日期 (Inspection Date) | 报告专题负责人/机构负责人日期 (Reported to Study Director /TFM Date) |
|---|---|---|---|
| 基于试验的检查 (Study-based inspection) | 试验方案 (Study Protocol) | 2023.05.17 | 2023.05.17 |
| | 试验设置（方案定义）(Study Set Up(Protocol Definition)) | 2023.05.23 | 2023.05.23 |
| | 给药操作 (Dose Administration) | 2023.05.25 | 2023.05.25 |
| | 阶段报告及试验记录 (Stage Report and Raw Data) | 2023.06.14-2023.06.15 | 2023.06.15 |
| | 病理报告及记录 (Pathology Report and Raw Data) | 2023.07.27 | 2023.07.27 |
| | 总结报告及试验记录 (Final Report and Raw Data) | 2023.07.31 | 2023.07.31 |

另外，根据本机构 SOPs 进行基于设施的检查。

In addition, facility-based inspections were conducted according to SOPs of the test facility.

签名（Signature）：邢清清　　日期（Date）：2023年07月31日

邢清清（Qingqing Xing），B.S.　　YYYY / MM/ DD

QA 检查员（Quality Assurance Auditor）

# List of Abbreviations and Definitions of Terms

| Abbreviations | Definitions |
|---|---|
| COA | Certificate of Analysis |
| D | Day |
| FDA | Food and Drug Administration |
| hr | Hour |
| hsTn I | High-Sensitivity Cardiac Troponin I |
| ICH | The International Council for Harmonisation of Technical Requirements for Pharmaceuticals for Human Use |
| SPF | Specific Pathogen Free |
| GLP | Good Laboratory Practice |
| NMPA (CFDA) | National Medical Products Administration |
| MTD | Maximal Tolerated Dose |

# Table of Contents

List of Abbreviations and Definitions of Terms ..... 5
Summary ..... 9
1. Introduction ..... 10
1.1. Study Title ..... 10
1.2. Study Number ..... 10
1.3. Objective ..... 10
1.4. Test Facility ..... 10
1.5. Sponsor ..... 10
1.6. Study Monitor ..... 10
1.7. Study Director ..... 10
1.8. Study Personnel ..... 10
1.9. Study Schedule ..... 11
1.10. Guidelines ..... 11
2. Test and Control Article ..... 11
2.1. Test Article ..... 11
2.2. Control Article ..... 11
2.2.1. Vehicle Control Article ..... 11
2.2.2. Negative Control Article ..... 12
2.3. Dose Formulation Preparation ..... 12
2.3.1. Preparation ..... 12
2.3.2. Storage ..... 12
2.3.3. Stability and Homogeneity of Dose Formulation ..... 12
2.4. Disposition of Remaining Dose Formulations and Test Articles ..... 12
2.5. Safety Precaution ..... 13
3. Test System ..... 13
3.1. Test Animals ..... 13
3.1.1. Species/Strain and Grade ..... 13
3.1.2. Number and Sex of Animals for Screening ..... 13
3.1.3. Number and Sex of Animals to be Used in the Study ..... 13
3.1.4. Body Weight and Age of Animals at Grouping ..... 13
3.1.5. Source and Production License No. ..... 13
3.1.6. Rationale for Choice of Species and Number ..... 13
3.1.7. Animal Identification ..... 13
3.2. Husbandry ..... 14
3.2.1. Quarantine/Acclimation ..... 14
3.2.2. Housing ..... 14
3.2.3. Environmental Conditions ..... 14
3.2.4. Food ..... 14
3.2.5. Water ..... 14
3.2.6. Bedding ..... 14
3.3. Pre-experiment Health Screening ..... 15
3.4. Randomization ..... 15
3.5. Study Design ..... 15

3.5.1. Rationale for Dose Selection ..... 15
3.5.2. Dose Levels ..... 15
4. Study Procedure ..... 15
4.1. Administration ..... 15
4.1.1. Route of Administration and Justification ..... 15
4.1.2. Dose Frequency and Justification ..... 16
4.1.3. Dose Site ..... 16
4.1.4. Observation Period ..... 16
4.1.5. Dose Speed ..... 16
4.1.6. Dose Volume ..... 16
4.2. Observation and Examination ..... 16
4.2.1. Mortality and Moribundity ..... 16
4.2.2. General Observation ..... 16
4.2.3. Body Weight ..... 16
4.3. Clinical Pathology ..... 17
4.3.1. Hematology and Coagulation ..... 17
4.3.2. Clinical Chemistry ..... 18
4.3.3. High-Sensitivity Cardiac Troponin I (hsTn I) ..... 18
4.4. Terminal Procedures ..... 19
4.4.1. Unscheduled Death ..... 19
4.4.2. Gross Necropsy ..... 19
4.4.3. Histopathological Examination ..... 19
4.5. Statistical Analysis ..... 20
4.6. Major Computer Systems ..... 20
5. Results ..... 20
5.1. Mortality and Moribundity ..... 20
5.2. General Observation ..... 21
5.3. Body Weight ..... 21
5.4. Clinical Pathology ..... 21
5.4.1. Hematology and Coagulation ..... 21
5.4.2. Clinical Chemistry ..... 21
5.4.3. High-Sensitivity Cardiac Troponin I (hsTn I) ..... 21
5.5. Histopathological Examination ..... 22
5.5.1. Macroscopic Findings ..... 22
5.5.2. Microscopic Findings ..... 22
6. Conclusion ..... 22
7. Archive ..... 22
Table 1 Mortality and Moribundity ..... 23
Table 2 General Observation ..... 25
Table 3 Summary of Body Weight ..... 30
Table 4 Summary of Hematology and Coagulation ..... 37
Table 5 Summary of Clinical Chemistry ..... 51
Table 6 Summary of High-Sensitivity Cardiac Troponin I (hsTn I) ..... 66
Appendix I The Certificate of Analysis of test article ..... 73
Appendix II The Certificate of Analysis of vehicle control article ..... 75

## Summary

In this study, the Sprague-Dawley (SD) rats were given 150 μg/animal dose of CD-801 injection via single intravenous injection. 0.9% sodium chloride injection for the negative control group was set. LNP for the vehicle control group was set to evaluate the toxicity of the delivery system. The dose volume of all groups was 3.0 mL/animal. Each group had a total of 10 animals, half male and female. The main toxicity assessment parameters included mortality/moribundity, general observation, body weight, hematology, coagulation, clinical chemistry and high-sensitivity cardiac troponin I. All surviving animals were grossly necropsied at the end of observation period (Day 15).

During the study, all animals survived to the scheduled necropsies. No test article-related effects were noted on general observation, body weight, and clinical pathology parameters (including hematology, coagulation and clinical chemistry). The detection of high-sensitivity cardiac troponin I, which is cardiac injury biomarker, showed no abnormalities. At the end of the observation period, no abnormal macroscopic findings or histopathological findings were noted in all animals.

Male and female animals in the test article group had shown slow weight gain since given a single dose, which was consistent with the vehicle control group. A transient slight decrease in ALB occurred on D4 (Day 4) after administration. The above changes were considered to be related to the test article but toxicologically insignificant due to small fluctuations.

In Summary, under the conditions of the study, SD rats were administered with CD-801 Injection via intravenous injection at the single dose of 150 μg/animal. All animals are well tolerated and no test article-related toxic effects were noted. Therefore, the maximal tolerated dose (MTD) of CD-801 Injection in Sprague-Dawley Rats via intravenous injection was 150 μg/animal
